# Neuregulin-1, a potential therapeutic target for cardiac repair

**DOI:** 10.3389/fphar.2022.945206

**Published:** 2022-08-31

**Authors:** Yan Wang, Jianliang Wei, Peng Zhang, Xin Zhang, Yifei Wang, Wenjing Chen, Yanan Zhao, Xiangning Cui

**Affiliations:** ^1^ First Clinical Medical School, Shandong University of Traditional Chinese Medicine, Jinan, Shandong, China; ^2^ Affiliated Hospital of Shandong University of Traditional Chinese Medicine, Jinan, Shandong, China; ^3^ Department of Cardiovascular, Guang’anmen Hospital, China Academy of Chinese Medical Sciences, Beijing, China

**Keywords:** cardiomyocyte proliferation, cardiac repair, cardiovascular disease, neuregulin-1 (Nrg1), therapeutic target

## Abstract

NRG1 (Neuregulin-1) is an effective cardiomyocyte proliferator, secreted and released by endothelial vascular cells, and affects the cardiovascular system. It plays a major role in heart growth, proliferation, differentiation, apoptosis, and other cardiovascular processes. Numerous experiments have shown that NRG1 can repair the heart in the pathophysiology of atherosclerosis, myocardial infarction, ischemia reperfusion, heart failure, cardiomyopathy and other cardiovascular diseases. NRG1 can connect related signaling pathways through the NRG1/ErbB pathway, which form signal cascades to improve the myocardial microenvironment, such as regulating cardiac inflammation, oxidative stress, necrotic apoptosis. Here, we summarize recent research advances on the molecular mechanisms of NRG1, elucidate the contribution of NRG1 to cardiovascular disease, discuss therapeutic approaches targeting NRG1 associated with cardiovascular disease, and highlight areas for future research.

## 1 Introduction

NRG1 (Neuregulin-1) belongs to the neuregulin family of growth factors responsible for cardiac development and cardiac protection from stress (Lin et al., 2020) and is a protein that plays a central role in cell signaling in the heart, mammary gland, and central nervous system (Falls, 2003; Mei and Nave, 2014). As a cardiac regeneration growth factor, NRG1 has attracted the attention of cardiovascular medicine. NRG1 has been shown to not only stimulate cardiomyocyte proliferation (Zhao et al., 1998; Bersell et al., 2009; D'Uva et al., 2015), but also act within angiogenesis, extracellular matrix remodeling, cardiomyocyte proliferation, stem-cell recruitment, and others, that improve cardiac function (Mendes-Ferreira et al., 2016; Rebouças et al., 2016; Arora et al., 2021). Together, these mechanisms promote myocardial repair and improvement of the cardiac function, that providing a novel molecular strategy targeting at regenerating the injured myocardium.

NRG1 is a powerful cardiovascular proliferation stimulant that is essential to the differentiation, proliferation, and expression of cardiac cells (Gassmann et al., 1995; Lee et al., 1995; Meyer and Birchmeier, 1995). NRG1 administration activates multiple signaling pathways to stimulate reentry and division of the adult cardiomyocyte cycle (D'Uva et al., 2015; Lin et al., 2015) and induce myocardial regeneration. Exogenous administration of NRG1 promotes the expression of genes related to the type of work (Zhu et al., 2010) and induces cardiomyocyte differentiation into cardiac conduction system cells (Rentschler et al., 2002), promotes stem cell differentiation into working-type cardiomyocytes (Suk Kim et al., 2003), and improves cardiac tissue function after pathological injury (Mendes-Ferreira et al., 2016; Kundumani-Sridharan et al., 2021; Sun et al., 2021), and are the most promising molecules for promoting cardiac repair under experimental conditions and clinical trials (Gao et al., 2010; Jabbour et al., 2011a; Polizzotti et al., 2015). Herein, this article summarizes the molecular mechanisms of NRG1 and its recent findings in cardiovascular biology, clinical applications in cardiovascular repair, and proposed areas of future research.

## 2 NRG1 and its regulation

NRG1’s regulatory mechanisms are complex and include a number of signalling, molecular, and metabolic pathways. Therefore, the molecular structure of NRG1, transcription regulation modes, and the interactions of signal pathways were summarized in this part.

### 2.1 Basic structure of NRG1

NRG1 is the most characteristic member of the neuregulin family (Huang et al., 2015a). The NRG1 gene is found on chromosome 8 in humans and mice and on chromosome 16 in rats (Falls, 2003; Chou and Ozaki, 2010). NRG-1 encodes 21 exons (Peles et al., 1992; Steinthorsdottir et al., 2004) and generates 31 potential protein isoforms (Mei and Xiong, 2008). Depending on the structure, NRG1 isoforms are classified into six types I-VI (Meyer et al., 1997; Steinthorsdottir et al., 2004). Although different isoforms of NRG1 are expressed in cells with different efficiencies, all can complete their signaling communication with ligands (Cote et al., 2005).

NRG1 consists of NH2-terminal ECD (extracellular structural domains), transmembrane structural domains, and highly conserved COOH-terminal ICD (intracellular structural domains) (Falls et al., 1993; Buonanno and Fischbach, 2001; Bao et al., 2004). NRG1-ECD binds to ErbB to activate ErbB signaling in target cells (Marchionni et al., 1993). It can be transferred to the nucleus and inhibit the expression of several apoptotic regulators (Bao et al., 2004). The interaction between ErbB and ECD in cells can occur after the release of NRG1-ECD or directly after the synthesis of isoforms lacking isoforms lacking transmembrane structural domains or after protein hydrolysis in the form of transmembrane precursors (Stonecypher et al., 2006; Yin et al., 2015). NRG1-ErbB interactions can also occur in cell-cell contact (Leimeroth et al., 2002). NRG1-ICD is required for NRG1 function *in vivo* (Liu et al., 1998), interaction of CRD (cysteine-rich domain) with ErbB receptors triggers signals required for CRD-NRG1 expression cells (Bao et al., 2003), and ectopic expression of NRG1 leads to NRG1-ICD dependent apoptosis (Grimm and Leder, 1997; Grimm et al., 1998).

**FIGURE 1:**
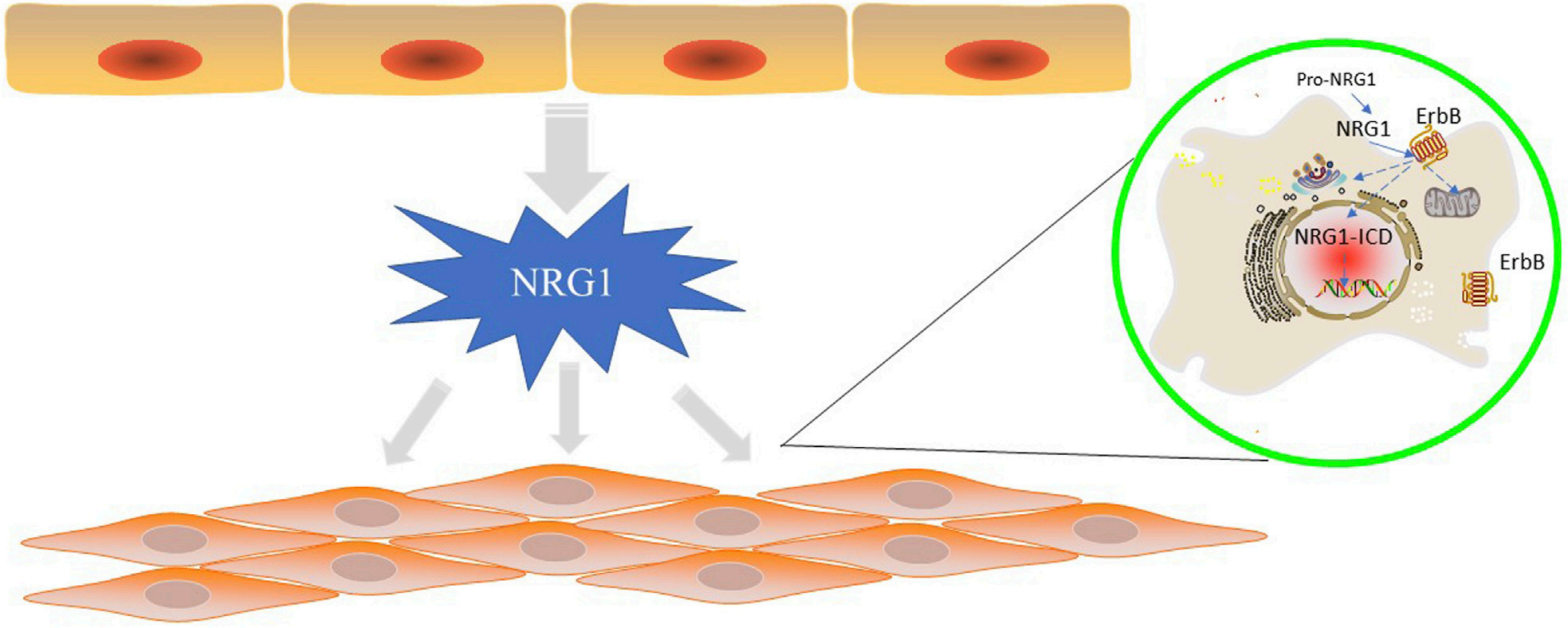
NRG1 is secreted by endothelial cells in the cardiovascular vessels and function in endothelial cells or cardiomyocytes by ErbB proteins. In “reverse signaling”, the C-terminal fragment of pro-NRG1 is generated by extracellular cleavage to release mature NRG1 and is further cleaved by γ-secretase to generate NRG1-ICD, which relocates to the nucleus to regulate gene transcription. In typical positive signaling, NRG1 binds to ErbB3 and ErbB4 ECD in differentiated cardiomyocytes and induces conformational changes, gaining higher affinity for other ErbB receptors. NRG1 ligand binding triggers homodimerization of ErbB4 and heterodimerization of ErbB2/3, ErbB2/4, ErbB3/4, followed by downstream signalling through the central pathway.

NRG1 is homologous to the EGF (epidemal growth factor) family (Marchionni et al., 1993; Garratt et al., 2000) and shares an EGF-like structural domain with all isoforms (Buonanno and Fischbach, 2001). The EGF-like structural domain is encoded by a common “core” exon that encodes the 5′-terminus of the EGF-like structural domain and is spliced with one of two different exons that encode the 3′-terminus of the EGF-like structural domain to produce different variants of the EGF-like structural domain (α and β) of NRG1, respectively (Buonanno and Fischbach, 2001; Falls, 2003). NRG1α is expressed at higher levels than NRG1β (Cote et al., 2005), while NRG1β having a higher affinity for ErbB4 than NRG1α (Plowman et al., 1993; Du et al., 2012). This differential may have important effects on the organism. Animal experiments have shown that NRG1β knockout mice are embryonically lethal (Meyer and Birchmeier, 1995; Kramer et al., 1996), whereas NRG1α knockout mice survive into adulthood with only significant defects in the alveolar development of the mammary lobe (Li et al., 2002). Even more important, NRG1α enhances cell adhesion molecule l1 expression in human glioma cells and promotes their migration as a function of malignancy (Zhao and Schachner, 2013). These suggest that NRG1β may play a more important role in survival than NRG1α. And another *in vitro* experiment on cardiac muscle cells further explored the effects of both on cardiac energy metabolism. NRG1β is a more potent activator of receptor phosphorylation and intracellular signaling than the NRG1α, and only NRG1β stimulated glucose uptake and protein synthesis (Cote et al., 2005). It means that only NRG1β are biologically active on cardiac myocytes although cardiac microvascular endothelial cells express multiple NRG1 isotypes (Cote et al., 2005) (Figure 1).

### 2.2 Regulation of NRG1

#### 2.2.1 Post-translational modifications

In “reverse signaling,” the C-terminal fragment of pro-NRG1 (NRG1 precursor) is generated by extracellular cleavage to release mature NRG1 and is further cleaved by γ-secretase to generate NRG1-ICD, which relocates to the nucleus to regulate gene transcription (Loeb et al., 1998; Wang et al., 2001). NRG1 expressed in the heart is a pro-NRG1 transmembrane protein that requires protease processing for activation (Cote et al., 2005). Earlier studies showed that the ADAM (a disintegrin and metalloproteinase) family of MMP (matrix metalloproteinases), specifically ADAM17 and ADAM19, play a role in the release of pro-NRG1 from the endothelial membrane (Montero et al., 2000; Shirakabe et al., 2001; Shi et al., 2003; Kurohara et al., 2004). Follow-up studies have shown that ADAM17 is the main protease involved in pro-NRG1 release, while ADAM19 does not play a role (Horiuchi et al., 2005). This indicates that other proteins that can activate pro-NRG1 may be involved, as the ADAM17-deficient mouse was expressed differently from the NRG1-deficient mouse in terms of heart defects in the embryo (Horiuchi et al., 2005). NRG1 mRNA expression and protein synthesis are mediated by neurohormones (such as angiotensin II, phenylephrine, endothelin 1) and the mechanical pressure (Lemmens et al., 2006). These factors stimulate the expression of mRNA of NRG1 by inhibiting the expression level of NRG1 (Lemmens et al., 2006). ENOS (endothelial nitric oxide synthesis) also regulates the expression of NRG1 expression, and recent studies have found that endothelial cell increases its expression of NRG1 when NO production is eliminated (Shakeri et al., 2021), and the two complement each other through the paracrine pathway.

#### 2.2.2 NRG1-ErbB signal pathway

NRG1 is produced mainly by endocardial vascular endothelial cells (Russell et al., 2014) and is an endogenous ligand of the ErbB family (Bouyain et al., 2005). NRG1 functioned through the receptor ErbB that all the biological effects of NRG1 were significantly reversed by the co-administration of NRG1 and ErbB inhibitor (Ding et al., 2021). The ErbB family consists of (ErbB1/EGFR), ErbB2 (HER2), ErbB3 (HER3) and ErbB4 (HER4) (Olayioye et al., 2000). All ErbB receptors are expressed on cardiac endothelial cells, fibroblasts and highly proliferative cells (Yarden and Sliwkowski, 2001), with ErbB3 and ErbB4 highly expressed on monocytes and cardiac macrophages (Carraway and Cantley, 1994; Campreciós et al., 2011), and ErbB2, ErbB4 on adult cardiomyocytes (Ma et al., 2016). The NRG1 gene encodes multiple isoforms of ErbB ligands (Pinkas-Kramarski et al., 1998; Buonanno and Fischbach, 2001). Although NRG-encoded protein isoforms by NRG differ greatly in their overall structure, the structural domain similar to EGF is sufficient to binding to receptors and trigger signals (Holmes et al., 1992).

NRG “forward” signaling, i.e., signaling from NRG-producing cells to NRG-responsive cells, leads to cellular responses, including stimulation or inhibition of proliferation, apoptosis, migration, differentiation, and adhesion (Yarden and Sliwkowski, 2001). In typical positive signaling, NRG1 binds to ErbB3 and ErbB4 ECD in differentiated cardiomyocytes and induces conformational changes (Jones et al., 1998), gaining higher affinity for other ErbB receptors (Kataria et al., 2019). NRG1 ligand binding triggers homodimerization of ErbB4 and heterodimerization of ErbB2/3, ErbB2/4, ErbB3/4 (Carraway and Cantley, 1994; Sweeney et al., 2001; Yarden and Sliwkowski, 2001), followed by downstream signaling through the central pathway (Xu et al., 2010; Fernandez-Cuesta et al., 2014). In some studies, the NRG1-ErbB pathway is believed to be a compensatory protective mechanism for cardiac injury (Bersell et al., 2009; Odiete et al., 2012; Dugaucquier et al., 2020). The NRG1-ErbB signaling axis is a critical mediator of cardiac development, and growing evidence supports a role for this system in the intricate cross-talk between the microvascular endothelium and myocytes in the adult heart.

#### 2.2.3 Signaling cascades

1) NRG1-Hippo-YAP pathway

NRG1-ErbB4 has been shown to strongly regulate the Hippo-YAP (Yes-associated protein) pathway (Haskins et al., 2014). Haskins et al. identified the transcriptional program induced by NRG1-ErbB4 signaling as the true molecular signature of the Hippo-YAP pathway by analyzing the expression of YAP-regulated genes in several functional readouts of the NRG1 activation pathway (Haskins et al., 2014). NRG1 administration stimulates re-entry and division of the adult cardiomyocyte cycle by triggering ErbB2/ErbB4-dependent inhibition of the Hippo-YAP signaling pathway (Bersell et al., 2009; D'Uva et al., 2015; Lin et al., 2015), increasing cardiomyocyte numbers rather than hypertrophic growth (D'Uva et al., 2015; Lin et al., 2015).

The components of the Hippo pathway are divided into two main categories: core kinase modules and transcriptional modules, of which the kinase modules include MSTs (mammalian sterile 20-like kinases), LATSs (large tumor suppressor kinases), SAV1 (salvador homolog 1), MOBs (mps one binder kinase activator proteins), and RASSFs (ras associated domain family), which are responsible for the repression of the transcriptional coactivator YAP and its homologue TAZ (transcriptional coactivator with PDZ binding motif). When the Hippo signaling pathway is activated, MST1/2 forms a complex with SAV1 that phosphorylates and activates the LATS1/2-MOB1 complex, which phosphorylates YAP and TAZ, and the phosphorylated YAP and TAZ pathway complexes are moved out of the nucleus, where they remain in the cytoplasm and are degraded by the protease system to lose transcriptional activity (Zhang and Re, 2017). This prevents their nuclear localization and thus inhibits cardiomyocyte proliferation (Heallen et al., 2011a; Dey et al., 2020). When the Hippo-YAP signaling pathway is inhibited, YAP dephosphorylates and translocates to the nucleus to bind to transcription factors such as TEAD (transcriptional coactivator with PDZ binding motif) to initiate the transcriptional process to promote mitosis (Lin and Pu, 2014). Therefore, outside the regeneration window, this suggests that modulation of the NRG1-Hippo-YAP pathways could unlock adult mammals’ ability to regenerate their hearts.

There is increasing evidence that YAP signaling interacts with PI3K (phosphatidylinositol-3-kinase)-AKT (a serine/threonine kinase) signaling to positively regulate cardiomyocyte cycle progression (Sudol, 2014; Lin et al., 2015). ErbB4 activates PI3K signaling in response to IGF (insulin-like growth factor) which plays a role in promoting cardiomyocyte growth and inhibiting fibrosis (MeiKim et al., 2013). YAP activates the IGF-PI3K signaling pathway in cardiomyocytes, leading to inactivation of GSK3β (glycogen synthase kinase 3β) and an increase in its downstream β-catenin content (Xin et al., 2011a). Furthermore, YAP can directly target the expression of Pik3cb (phosphatidylinositol-4,5-bisphosphate 3-kinase catalytic subunit beta), the catalytic submitter of PI3K, to activate the PI3K-AKT pathway (Lin et al., 2015) to indirectly promote β-catenin activity.

2) NRG1-PI3K-AKT pathway

NRG1-ErbB4 can inhibit through PI3K-AKT pathway macrophages to reduce myocardial inflammation and fibrosis (Vermeulen et al., 2017), while inhibiting cardiac endoplasmic reticulum stress to reduce reperfusion injury (Fang et al., 2017). NRG1 maintains cardiac troponins by the ErbB2-PI3K pathway, which may lessen doxorubicin-induced cardiac dysfunction (Bian et al., 2009). Together, these studies confirmed that the activation of the NRG1-ErbB pathway plays a protective role in myocardial injury and may be involved in cell apoptosis and differentiation, as found in studies that NRG1/ErbB2 regulates cell differentiation and apoptosis through the PI3K/Akt pathway (Hong et al., 2021; Lu et al., 2021; Mahiny-Shahmohammady et al., 2022). *In vitro*, NRG1 treatment up-regulated ErbB3 phosphorylation, and increased the expression of PI3K and phosphorylation-AKT (Meng et al., 2021) to modulate the activation of PI3K-AKT pathway. In conclusion, NRG1 can act on ErbB2, ERBB3 and ERBB4, respectively activates PI3K-AKT pathway.

EGF induces YAP nuclear accumulation through PI3K-dependent blockade of YAP phosphorylation. EGF triggers the rapid translocation of YAP into the nucleus along with YAP dephosphorylation, both of which depend on LATS. EGF receptor inhibits the Hippo pathway through activation of PI3K and PDK1, but independent of AKT activity. The entire Hippo core complex dissociates in response to EGF signaling in a PI3K-PDK1-dependent manner, leading to inactivation of LATS, dephosphorylation of YAP, and YAP nuclear accumulation and transcriptional activation of its target gene. In turn, activated YAP induces transcription of amphiregulin (Zhang et al., 2009) and promotes positive feedback regulation of Hippo signaling by activation of EGF receptor.

3) other related pathways

Based on the available literature, it is known that NRG1 can regulate cardiac physiopathological processes through the interaction between Hippo pathway, Wnt pathway, ERK (extracellular signal-regulated kinase) pathway, RISK pathway and mTOR (rapamycin) pathway.

NRG1-ErbB2 is involved in the negative regulation of cardiomyocyte autophagy by activating the PI3K/PKB/mTOR and MAPK (monophosphate-activated protein kinase)/ERK/mTOR pathways (An et al., 2013; Surviladze et al., 2013). Interestingly, recent studies have found that ErbB2 signaling can lead to YAP to promote cell proliferation in an ERK-dependent manner rather than Hippo (Aharonov et al., 2020), and can also indirectly promote the onset of cardiac regeneration through interactions of the AKT and GSK3β/β-catenin pathway (D'Uva et al., 2015). For example, inactivation of cardiac SAV not only inhibits the Hippo pathway, releasing YAP into the nucleus for direct interaction with β-catenin (Heallen et al., 2013) to enhance its transcriptional activity, but also upregulates the classical Wnt pathway (Heallen et al., 2011b) to act on target genes to promote cell mitosis process.

It has been demonstrated that NRG1 induces upregulation of ligand protein expression to promote the differentiation of embryonic stem cells into working cardiomyocytes via MEK (mitogen-extracellular activated protein kinase)-ERK (Zhu et al., 2010; Wang and Huang, 2014). In cardiac transplantation, rhNRG1 (recombinant human NRG1) attenuates left ventricular remodeling and myonodal disorders by up-regulation of the RISK pathway (Liu et al., 2006; Gao et al., 2010) to create conditions to maintain donor heart function. Due to the interaction between signaling pathways and other signal crosstalk between cells, the signaling cascades of NRG1 is still unclear, and further research is still needed.

## 3 NRG1 with cardiovascular diseases

**FIGURE 2:**
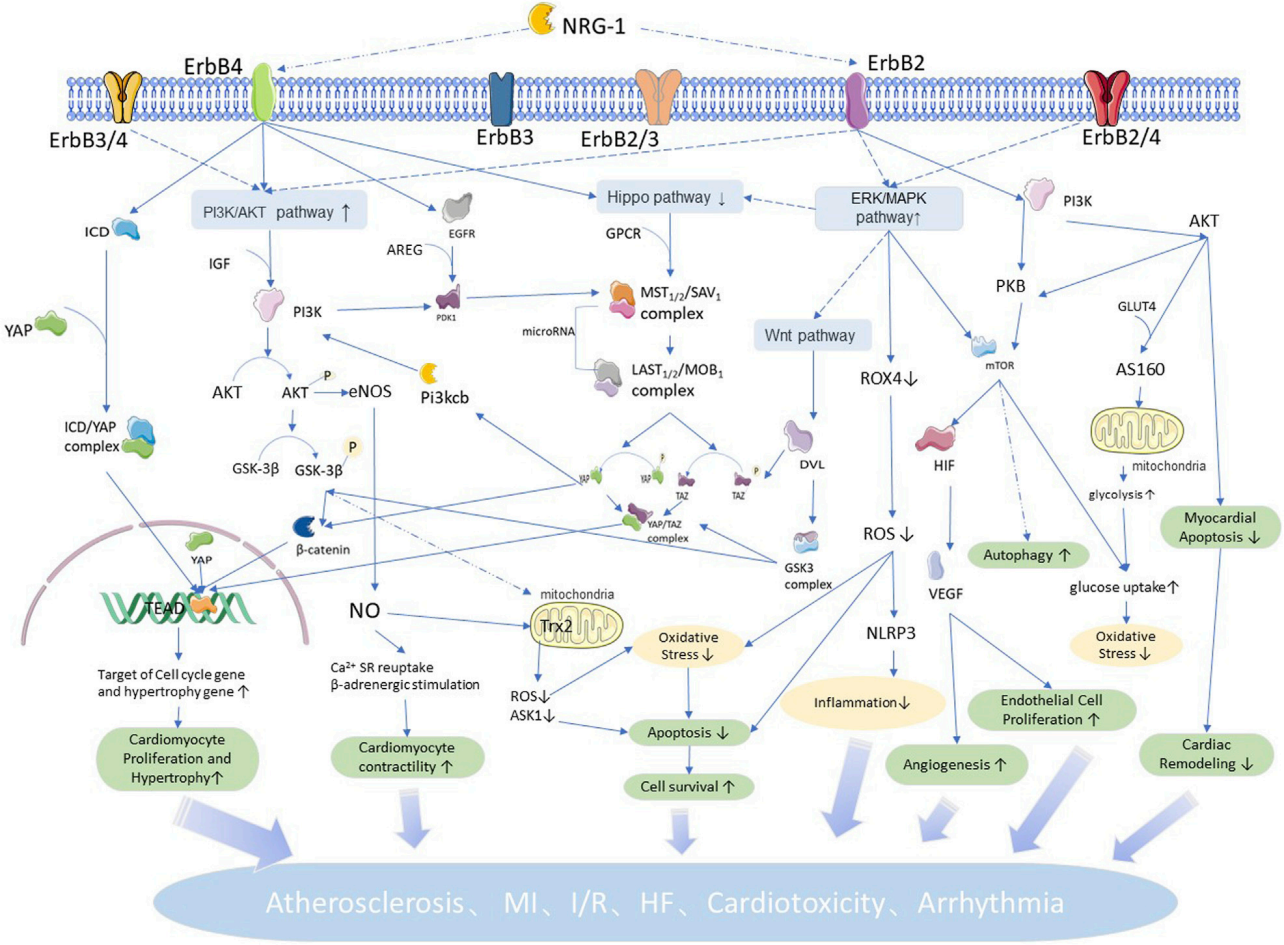
NRG1 related pathways and roles in cardiovascular: NRG1 mediated by ErbB4 regulates cell proliferation and hypertrophic growth by coordinating the Hippo pathway, and regulates apoptosis and proliferation, oxidative stress, cell autophagy and other physiopathological processes by the P13K/AKT pathway. NRG1 mediated by ErbB4 regulates cellular inflammatory response and vascular proliferation by coordinating the ERK/MAPK pathway, and the Wnt pathway is involved in regulating cardiac and vascular neogenesis. Meanwhile, the interaction between signaling pathways makes each physiopathological process intricate and complex, increasing with each other and participating in NRG1 to regulate the development of cardiovascular diseases.

The downstream signaling of NRG1 acts mainly in the ventricular wall, heart valves and conduction system, and microvasculature (Rupert and Coulombe, 2015). In this section, we summarize the physiological and pathological roles of NRG1 in cellular and cardiovascular tissues to fully illustrate the important role of NRG1 in the development of cardiovascular diseases. Novel research shows that NRG1 plays an important role in the occurrence and development of cardiovascular diseases, such as atherosclerosis, MI/IR, HF, cardiotoxicity, and arrhythmia. We summarize the role of NRG1 in cardiovascular diseases (Figure 2) in conjunction with the related pathways. In addition, animal experiments and clinical studies related to the treatment of cardiovascular diseases by NRG1 are summarized in Table 1.

**TABLE 1:** The role of NRG1 in cardiovascular disease.

Disease	NRG1 expression	Human studies	Examples of *in vivo* studies
			rhNRG1 produced global improvements in cardiac function in a canine model of pacing-induced heart failure. (Liu et al., 2006)
			rhNRG1 can significantly improve left ventricular remodeling and cardiac function in the failing heart, this beneficial effect is related to reducing mitochondrial dysfunction, myocyte apoptosis and oxidative stress. (Guo et al., 2012)
			rhNRG1 treatment attenuates pulmonary arterial and right ventricular remodelling and dysfunction in a rat model of monocrotaline-induced pulmonary arterial hypertension, and has direct anti-remodelling effects on the pressure-overloaded right ventricular. (Mendes-Ferreira et al., 2016)
Heart failure	rhNRG1	Both phase 1 and 2 trials have been completed finding sustained improvement in LVEF in systolic heart failure patients at 90-days follow-up. A phase 3 trial is currently on going. (Gao et al., 2010; Jabbour et al., 2011b)	#FF0000
			NRG1 induces cardiac hypertrophy and impairs cardiac performance in post-myocardial infarction rats. (Zurek et al., 2020)
Atherosclerosis	rhNRG1	The effect of job strain on early atherosclerosis is dependent on NRG1 genotype in men. (Hintsanen et al., 2007)	rhNRG1 treatment induced systemic activation of ErbB2 and ErbB4 receptors in both heart and kidneys and prevented left ventricular dilatation, improved left ventricular contractile function, and reduced atherosclerotic plaque size. (Vandekerckhove et al., 2016)
			#FF0000
			rhNRG1 improved cardiac performance, with the survival benefits in the ischemic model being additive to those of angiotensin-converting enzyme inhibitor therapy. (Liu et al., 2006)
			cMLCK is a downstream effector of rhNRG-1 involved in rhNRG1-induced cardiac function improvement. (Gu et al., 2010)
Myocardial infarction	rhNRG1	A single sample determination of NRG1β at emergency departments presentation is not predictive of a final diagnosis of MI or acute coronary syndrome. (Yiadom et al., 2016)	#FF0000
			Overexpression of ErbB4 rejuvenates aged mesenchymal stem cells and enhances angiogenesis via PI3K/AKT and MAPK/ERK pathways. (Liang et al., 2019)
			The concentration of NRG1 was rapidly upregulated after myocardial IR. NRG1 preconditioning with an optimal concentration of 4 μg/kg has cardioprotective effects against IR injury through a PI3K/Akt-dependent mechanism *in vivo*. (Fang et al., 2010)
Ischemia reperfusion	NRG1	None	#FF0000
			Remote ischemic perconditioning play an anti-remodeling and anti-inflammatory role by acting of the NRG1/ErbB3 signal. (Pilz et al., 2019)
Cardiotoxicity	Engineered bivalent NRG1β	None	Engineered bivalent NRG1β promotes cardiomyocyte survival and improves cardiac function. Given the reduced proneoplastic potential of Engineered bivalent NRG1β versus NRG, engineered bivalent NRG1β has translational potential for cardioprotection in patients with cancer receiving anthracyclines. (Jay et al., 2013)

cMLCK, cardiac myosin light chain kinase; IR, ischemia reperfusion; rhNRG1, human recombinant NRG1.

### 3.1 NRG1 and atherosclerosis

#### 3.1.1 NRG1 and cell aging

Cellular senescence is driven by chronic inflammation and oxidative stress. The senescence of EC (endothelial cell) and VSMC (vascular smooth muscle cell) can cause atherosclerosis (Minamino and Komuro, 2008; Nakano-Kurimoto et al., 2009; Wang and Bennett, 2012), and cellular senescence and SASP (senescence-associated secretory phenotype) play an important role in cardiovascular aging and disease (Gorenne et al., 2006; Minamino and Komuro, 2007; Sikora et al., 2011). Cells can enter a state of senescence caused by DNA damage due to intrinsic and extrinsic stressors, called “stress-induced premature senescence” (SIPS) (Correia-Melo et al., 2014). Oxidative stress and hyperglycemia are the two main factors that contribute to SIPS (Gorenne et al., 2006; Minamino and Komuro, 2007; Sikora et al., 2011).

NRG1 is known to have improved glucose metabolism (Pentassuglia et al., 2016) and antioxidant (Wang et al., 2021) effects, so it may have anti-SIPS potential. Data suggests that NRG1 significantly inhibits stress-induced premature senescence in vascular cells *in vitro* and in the aorta of diabetic mice *in vivo* (Shakeri et al., 2018), and application of rhNRG-1 for 9 weeks to type 1 diabetic mice attenuates diabetes-induced vascular senescence (Shakeri et al., 2018). Consistently, deficiency of the NRG1 receptor ErbB4 induced cellular senescence both *in vitro* and *in vivo* (Shakeri et al., 2018), and diabetes induced significantly more vascular senescence in mice lacking the ErbB4 receptor in SMC compared to wild-type littermates (Shakeri et al., 2018), suggesting the efficacy of NRG1 in anti-cellular senescence. The effects of NRG1 on cell proliferation and apoptosis have been well described (Yarden and Sliwkowski, 2001; Xu et al., 2014), but the effects of NRG1/ErbB signaling on cellular senescence remain to be elucidated.

#### 3.1.2 NRG1 and endothelial cell damage

Previous experiments have demonstrated that NRG1 prevents foam cell formation in primary cultured human monocyte-derived macrophages (Xu et al., 2009). Also, chronic infusion of NRG1β into ApoE^−/−^ mice inhibited macrophage infiltration in the arterial wall (Xu et al., 2009), it reduced endocytosis of acetylated LDL (low density lipoprotein) and cholesterol ACAT-1 (acyltransferase-1) activity, and it increased cholesterol efflux from human macrophages to apolipoprotein A1 (Xu et al., 2009). This again confirms the inhibitory effect of NRG1β on atherosclerotic lesions. Co-incubation of VSMCs with norepinephrine and oxidized LDL inhibited NRG1 expression and induced proliferation and phenotypic transformation of VSMCs (Jiao et al., 2008). NRG1 reduces proliferation and migration of neointima formation after vascular injury in rats by reducing VSMCs (Clement et al., 2007). These findings suggest a negative correlation between NRG1 expression and proliferation in VSMCs. Stimulation of ErbB3 by NRG1β has been reported to inhibit EC proliferation (Amin et al., 2006), and ErbB3 may be a potential therapeutic target of the NRG1-ErbB pathway against atherosclerosis.

#### 3.1.3 NRG1 and vascular stress

In addition, the NRG1/ErbB signaling system is essential for the response to vascular stress (Parodi and Kuhn, 2014). NRG1 has anti-adrenergic effects on the cardiovascular system (Lemmens et al., 2004) leading to vasodilation. It was shown that microinjection of NRG into the ventral lateral aspect of the medulla oblongata, a major vasodilatory center, reduced blood pressure in rats (Matsukawa et al., 2013). NRG1β is an activator of nicotinic acetylcholine receptors, which may improve the atherosclerotic vascular load for cardioprotective effects by balancing the autonomic nervous system (Okoshi et al., 2004).

### 3.2 NRG1 and myocardial infarction

#### 3.2.1 NRG1 and oxidative stress

Myocardial infarction is characterized by a peak in cardiomyocyte death on the first day (Whelan et al., 2010; Del Re et al., 2019). The latest research shows that no association with other variables, NRG-1 plasma levels decreased significantly following PCI/RIC and remained decreased up to 1 month following AMI (Haller et al., 2022). ROS (Reactive oxygen species) generated in cardiac ischemia-reperfusion induce NRG1β/ErbB4 activation (Kuramochi et al., 2004a), implying that NRG1β/ErbB4 signaling regulates myocardial injury in an environment of oxidative stress. NADPH oxidase 4 (NOX4) is the major ROS synthesizing enzyme in cardiac tissue (Matsushima et al., 2014). Previous experiments have demonstrated that NRG1 can inhibit NOX4 activity by activating the ERK1/2 (Wang et al., 2018) pathway in myocardial reperfusion injury, thereby downregulating ROS production (Caja et al., 2009). Specific overexpression of ErbB2 in young rat hearts induces the expression of antioxidant genes such as mitochondrial GPx1 (glutathione peroxidase 1) and catalase (Belmonte et al., 2015). NRG1 can inhibit hydrogen peroxide by acting ErbB4-Akt signaling or by direct activation of paracrine signaling by ErbB4 independent of NRG in adult rat ventricular myocytes (Kuramochi et al., 2004a), thus inhibiting hydrogen peroxide stimulated adult rat ventricular myocytes apoptosis (Kuramochi et al., 2004a) to achieve self-protection. Thus, NRG1 exerts an anti-apoptotic effect on myocardium by coordinating the oxidative stress process.

#### 3.2.2 NRG1 and myocardial inflammatory injury

AMI (acute myocardial infarction) induces blood leukocytosis, which is negatively correlated with patient survival (Jiang et al., 2022). Excessive inflammation in endangered myocardial regions induced by MI can lead to increased cardiomyocyte death through pro-apoptotic signaling pathways and further cardiac remodeling (Nahrendorf et al., 2010; Griffiths et al., 2017). NRG1 receptor epidermal growth factor receptors ErbB2 and ErbB4 were expressed on cardiac fibroblasts in the infarct area. Systemic blockade of ErbB function in MI model mice enhanced senescence and apoptosis of cardiac fibroblasts and exacerbated inflammation (Shiraishi et al., 2022).

It has been shown that NRG1 can reduce ROS production through ERK1/2 inhibition of NOX4 and inhibit the NLRP3/caspase-1 pathway, thereby attenuating myocardial oxidative damage and inflammatory responses in MI (Wang et al., 2021). NRG1-mediated NOX4 inhibition is dependent on ERK1/2 activation (Wang et al., 2021). NOX4-mediated ROS production promotes activation of NLRP3 inflammatory vesicles (Abais et al., 2015; Moon et al., 2016), stimulates upregulation of NLRP3 inflammatory vesicles (Kawaguchi et al., 2011; Sandanger et al., 2013), and promotes activation of the inflammatory response by the organism. In a model of septic cardiomyopathy, NRG1 can inhibit NLRP3 inflammatory vesicles (Abais et al., 2015). NRG1β has also been reported to reduce the pro-inflammatory response by attenuating COX-2 (cyclooxygenase 2) expression in monocyte U937 cells (!!! INVALID CITATION) and can also exert anti-inflammatory antioxidant effects in myocardial tissues and cells by activating AKT/eNOS pathways (Kuramochi et al., 2004a; Timolati et al., 2006; Brero et al., 2010). The role of NRG1 in reducing inflammatory damage in cerebral IR has been previously reported (Wu et al., 2015). However, studies on the anti-inflammatory effects of NRG1 in myocardial tissue are limited.

#### 3.2.3 NRG1 and energy metabolism

Under stress conditions such as insufficiency and stress overload, lipid metabolism not only cannot meet the heart’s energy needs, but also exacerbates oxidative stress (Opie, 1976), with the heart increasing glucose uptake and decreasing the consumption of fatty acids to maintain the high energy demands required for sustained contraction (Shao and Tian, 2015; Riehle and Abel, 2016; Szablewski, 2017). Under such stressful conditions, myocardial endothelial cells also release NRG1 as a cardioprotective factor (Kuramochi et al., 2004a; Hedhli et al., 2011). NRG1-ErbB2 signaling has been found to induce cardiomyocytes in the border region of the injured heart to dedifferentiate and shift their metabolism from oxidative phosphorylation to glycolysis in zebrafish, as evidenced by the decrease in mitochondrial genes and the increase in glycolytic genes (Honkoop et al., 2019). NRG1 improves the pharmacokinetic properties and produces metabolic benefits by reducing the double inhibition of hepatic gluconeogenesis and cholesterol intake in obese mice (Zhang et al., 2018). These results indicate that NRG1 promotes energy metabolism in zebra fish, which maintain regeneration, and in mammals, which significantly reduce regeneration after birth. The activation of the NRG1-ErbB2 signal helps to reduce glucose consumption after heart damage and promote the use of glucose energy. RhNRG1 increases glucose uptake by neonatal rat ventricular myocytes through ErbB2/ErbB4 and PI3K (Liu et al., 2006). In the adult heart, glucose uptake by cardiac myocytes is believed to be primarily achieved by the glucose transporter protein GLUT4 (glomerular glucose transporter 4) (Shao and Tian, 2015; Riehle and Abel, 2016). In a neonatal rat ventricular myocyte model, similar to insulin, rhNRG1 can induce glucose uptake by activating the PI3K-Akt-AS160 (Akt substrate of 160 kDa) pathway and GLUT4 translocation, and unlike insulin, the rhNRG1-induced effects are not mediated by insulin recptorsubstrate proteins (Liu et al., 2006). RhNRG-1 has been shown to enhance glucose uptake by neonatal rat ventricular myocyte through a mechanism associated with the ErbB2/ErbB4, PI3K-Akt (Pentassuglia et al., 2016).

The mammalian target of mTOR regulates human cardiac protein synthesis and energy metabolism (Laplante and Sabatini, 2012; Saxton and Sabatini, 2017). MTORC1 activity, a complex of mTOR, is associated with protein synthesis and physiological hypertrophy, and mTORC2, another complex, regulates glucose uptake (Kumar et al., 2008; Liu et al., 2014; Sato et al., 2014). NRG1β has a direct and specific stimulatory effect mediated by mTOR in the cardiac myocyte signaling cascade with direct and specific stimulatory effects (Pentassuglia et al., 2016). ErbB receptor activity is associated with mTOR signaling, and mTOR inhibitors improve the outcome of ErbB2-positive breast cancer (Vicier et al., 2014). Although mTOR inhibition appears to have therapeutic value in cancer, cardiomyocyte mTORC1 deficiency leads to cardiac dysfunction in mice (Zhang et al., 2010; Shende et al., 2011). In a rat neonatal cardiomyocyte model, NRG1/ErbB signaling enhances glucose uptake and protein synthesis, but NRG1β-induced mTORC1 plays a minor role in protein synthesis, while mTORC2 appears to be independent of glucose uptake (Pentassuglia et al., 2016).

#### 3.2.4 NRG1 and cell multiplication

Hypertrophic growth, multinucleation, and polyploids are characteristics of cardiovascular cells in mammalians, and are the underlying cause of changes in cardiac cell growth before and after birth (Ikenishi et al., 2012; Tane et al., 2015). Cardiomyocytes undergo this process rapidly in infarcted and failing hearts in response to growth stimuli, leading to an increase in the number of multinucleated and polyploid cells in damaged myocardial tissue (Herget et al., 1997; Tevzadze et al., 2005; Stephen et al., 2009; Lázár et al., 2017; Kajstura et al., 2010), and cardiomyocytes grow hypertrophically without being able to achieve a quantitative increase. Furthermore, as the heart ages and the myocardium is released from the cell cycle, Hippo activity increases significantly, while YAP activity decreases considerably (Heallen et al., 2013; von Gise et al., 2012) and cell proliferation is endogenously inhibited. In recent years, clinical trials of the new approach to stem cell transplantation have shown limited success, but long-term effectiveness remains unknown (Wei et al., 2019; Hotham and Henson, 2020; Schwach et al., 2020). NRG1 was found to promote mitosis through ErbB (D'Uva et al., 2015) for efficient cell proliferation.

In mice, NRG1 administration has been shown to induce proliferation and cardiac regeneration in adult cardiomyocytes (Bersell et al., 2009). In fact, transient induction of ErbB2 signaling in juvenile and adult mice cardiomyocytes was sufficient to strongly induce cardiomyocyte dedifferentiation and proliferation and to trigger cardiac regeneration after myocardial infarction (D'Uva et al., 2015). In zebrafish, NRG1 is dramatically induced in perivascular cells after cardiac injury, and inhibition of its co-receptor ErbB2 disrupts the proliferative response of cardiomyocytes to injury (Gemberling et al., 2015). Following ischemic heart injury in adolescents or adults, transient expression of ErbB2 similarly triggers cardiomyocyte dedifferentiation, proliferation, neointima formation, and cardiomyocyte redifferentiation, which together lead to anatomical and functional regeneration (Lee et al., 1995; Lai et al., 2010; Liu et al., 2010; Staudt et al., 2014). This provides a definitive clinical experimental basis for the ability of NRG1-ErbB2 to effectively promote cell proliferation. Furthermore, NRG1-ErbB4 has been shown to induce mononuclear cardiomyocyte proliferation without affecting the level of apoptotic cell death (Zhu et al., 2010). These evidences suggest the feasibility of NRG1-ErbB pathway expression for myocardial regeneration as well as a clear value for clinical studies. However, as the organism matures, the cardiac level of ErbB2 decreases and its mitogenic promoting effect is significantly weaker in late postnatal development and adulthood than in the neonatal period (D'Uva et al., 2015; Polizzotti et al., 2015; Aharonov et al., 2020). Therefore, the strategy of combining NRG1 with ErbB2 overexpression or ErbB2-inducible factors should be further explored.

In the NRG1-Hippo-YAP signaling axis, deletion of YAP in the embryonic heart prevents cardiomyocyte proliferation and leads to myocardial dysplasia (von Gise et al., 2012; Xin et al., 2011b). In contrast, deletion of Sav1 or LST1/2, upstream negative regulators of YAP, and cardiomyocyte-specific hyperactivation of YAP increases embryonic cardiomyocyte proliferation (Heallen et al., 2011a; Heallen et al., 2013; von Gise et al., 2012; Xin et al., 2013). However, these experiments induce adult cardiac regeneration by inducing early cardiomyocytes with strong regenerative capacity, with limited clinical operability and application. Also, in this pathway, its downstream effector YAP/TAZ complex is overactivated in human cancers and is a hallmark of cancer. Therapeutic YAP/TAZ activation may contribute to the development of cancer (Harvey et al., 2013; Janse van Rensburg et al., 2018). The ability to harness the regenerative power of YAP/TAZ in a safe manner is critical for clinical application.

#### 3.2.5 NRG1 and angiogenesis

NRG1 is also a proangiogenic factor that plays an important role in angiogenesis and arteriogenesis after ischemic injury (Hedhli et al., 2012). Myocardial ECs upregulate NRG1 release during ischemic injury (Fukazawa et al., 2003; Hedhli et al., 2011). The receptor for NRG1, ErbB2, was found to be expressed only in ECs, but not in cardiomyocytes from adult mouse hearts (Kundumani-Sridharan et al., 2021). NRG1-ErbB2 can regulate angiogenesis not only through the activation of HIF-1α (hypoxia-inducible factor-1α) via the PI3K/AKT/mTOR pathway (Karar and Maity, 2011), but also through paracrine up-regulation of VEGF to induce angiogenesis (Xu et al., 2010). In a study by Kundumani Sridharan (Kundumani-Sridharan et al., 2021) et al., it was shown that NRG1β-ErbB2 signaling also protects ECs through the ErbB2/4-cSrc-NO axis and the ErbB2/4-ATG5-Trx2 (thioredoxin-2) axis. Although it is difficult to perform pro-angiogenic therapy after AMI (Grines et al., 2002; Hedman et al., 2003; Henry et al., 2003; Wu et al., 2021), proangiogenic therapy still has the potential to benefit patients in the acute phase of IR (Wu et al., 2021).

NRG1 regulates endothelial progenitor cell biology by affecting their survival and differentiation capacity (Safa et al., 2011; Hedhli et al., 2012). EPDCs (epicardial-derived cells) are transformed from epicardial progenitor cells and develop into cardiac fibroblasts and VSMCs (Acharya et al., 2012; Katz et al., 2012), which are essential for myocardial and coronary vascular development (Smits et al., 2018). Consecutive genetic and pharmacological studies have also shown that the arrest of fibroblast differentiation is mediated by YAP, which controls both the composition of ECM (extracellular matrix) and vascular remodeling during cardiac development (Xiao et al., 2018). Although the role of the NRG1-Hippo-YAP pathway in noncardiomyocytes remains less clear, these findings suggest that the NRG1-Hippo-YAP pathway is required for cardiac fibroblast differentiation and coronary vascular development.

### 3.3 NRG1 and ischemia reperfusion

The ischemic and hypoxic myocardial microenvironment leads to myocardial injury, induces an inflammatory response (Li et al., 2021), and initiates self-repair processes such as apoptosis and autophagy (Steffens et al., 2020). In the endothelium, IR injury leads to activation of EC (i.e., expression of pro-inflammatory molecules), increased permeability and edema, and impaired vasodilation (Wan and Rodrigues, 2016; Colliva et al., 2020; Wagner and Dimmeler, 2020). In turn, endothelial injury and dysfunction promote vasoconstriction, thrombosis, and coronary occlusion, leading to cardiac inflammation and injury, adverse cardiac remodeling, and hypertrophy (Prabhu and Frangogiannis, 2016; Gibb et al., 2020; Erkens et al., 2021).

NRG1 preconditioning protects the heart against ischemia/reperfusion injury through a PI3K/Akt-dependent mechanism (Fang et al., 2010). In Langendorff analyzes, the ErbB4 inhibitor suppressed the phosphorylation of ErbB4 and the RISK pathway and aggravated myocardial edema and fiber fracture, thereby inhibited the cardioprotective effects of NRG1 (Wang et al., 2018). For assessment of downstream signals, the PI3K inhibitor and the MEK inhibitor suppressed the phosphorylation of AKT and ERK1/2 respectively and abolished the cardioprotective effects induced by NRG1 (Wang et al., 2018). Another experiment further confirmed this idea, and proposed that NRG1 might protect the heart from IR damage by inhibiting endoplasmic reticulum stress through the PI3K-Akt pathway (Fang et al., 2017). Endothelial colony-forming cells were capable of actively releasing NRG1, which, in turn, reduced apoptosis and increased the proliferation of human pluripotent stem cell-derived cardiomyocytes via the PI3K/Akt signaling pathway (Hong et al., 2021), while transcriptional silencing of NRG1 abrogated these cardioprotective effects. Moreover, endothelial colony-forming cells are uniquely suited to support human pluripotent stem cell-derived cardiomyocytes, making these progenitor cells ideal for cardiovascular regenerative medicine (Hong et al., 2021).

RIPC (remote ischemic preconditioning) is an interventional procedure in which multiple short-cycle IR cycles applied in a remote vascular bed can prevent IR injury. The endothelial NRG1β-ErbB2 signaling pathway has been shown to have a good protective effect in RIPC-induced cardiac injury (Hedhli et al., 2011; Lemmens et al., 2011; Parodi and Kuhn, 2014; Kundumani-Sridharan et al., 2021). RIPC rescues the loss of mitochondrial Trx2 mediated by IR through NRG1β-dependent protection against endothelial ErbB2 loss in mice is an important previously unrecognized mechanism of RIPC-mediated protection against MI (Kundumani-Sridharan et al., 2021). Trx2 has been shown to inhibit mitochondrial ROS generation and ASK1 (apoptosis signal-regulated kinase 1) to maintain cardiac function (Huang et al., 2015b). NO is known to alter autophagy and endothelial cell NO production may inhibit cardiomyocyte autophagy leading to loss of Trx2 (Sarkar et al., 2011). Loss of Trx2 in cardiomyocytes leads to activation of ASK1 and promotes apoptosis in cardiomyocytes (Huang et al., 2015b). Endothelial ErbB2 reduces ASK1-mediated apoptosis in cardiac tissue by mediating the phosphorylative inactivation of ATG5 (autophagy related 5), thus inhibiting Trx2 degradation in mitochondria (Kundumani-Sridharan et al., 2021). Up-regulation of the ErbB2/4-ATG5-Trx2 pathway protects against cardiomyocyte apoptosis (Nonn et al., 2003; Huang et al., 2015b), providing a new therapeutic idea against myocardial apoptosis. Although NRG1 has been shown to have a beneficial role in cardiac function, circulating NRG1 levels correlate with the severity of HF (Ky et al., 2009), which is not related to the ability to repair damaged myocardium.

In the NRG1-Hippo-YAP signaling axis, inhibition of Hippo pathway expression can somewhat reduce the level of apoptosis in peri-infarct cardiomyocytes and reduce the expansion of infarct size. YamamotoYang et al. (2003) demonstrated in myocardial infarction model mice experiments that MST1 was significantly activated upon apoptosis and that cardiac-specific overexpression of MST1 transgenes activated mouse apoptosis-associated proteins caspase3 and caspase9 in cardiac tissues, resulting in increased apoptosis in cardiomyocytes (Odashima et al., 2007). On the contrary, specific knockdown of MST1 in their cardiac tissues significantly improved apoptosis in mice (Odashima et al., 2007). Cardiac-specific overexpression of MST1 in mice leads to progressive deterioration of cardiac function (Yamamoto et al., 2003), which may be the result of increased myocardial oxygen consumption and apoptosis due to elevated wall pressure.

### 3.4 NRG1 and hart failure

#### 3.4.1 NRG1 with myocardial hypertrophy and myocardial fibrosis

During the healing process of heart, damaged myocardial tissue undergoes myocardial hypertrophy, myocardial fibrosis, and ventricular remodeling, ultimately leading to myocardial hyposystolic function and heart failure (Wang et al., 2017a; Lindsey et al., 2018). In cases of heart failure, NRG1/ErbB signaling is significantly impaired (Galindo et al., 2014; Gupte et al., 2017), and administration of NRG1 attenuates the progression of HF, inhibits myocardial fibrosis and apoptosis, and reduces oxidant-producing enzymes (Gupte et al., 2017). Conditional knockout of ErbB2 in mice with accelerated heart failure after stress overload or adriamycin injury (Crone et al., 2002; Ozcelik et al., 2002) showed that the NRG/ErbB pathway acts as a stress response signal between microvascular endothelial cell and myocytes to maintain cell survival and cardiac function.

M2-like macrophage-mediated regulation of NRG1/ErbB signaling has a substantial effect on fibrotic tissue formation in the infarcted adult mouse heart and is critical for suppressing the progression of senescence and apoptosis of cardiac fibroblasts (Shiraishi et al., 2022). A recent study showed that the NRG1β-ErbB4 pathway also inhibits macrophage activation and reduces myocardial hypertrophy and fibrosis (Vermeulen et al., 2017). The molecular mechanism underlying the regulation of fibrotic tissue formation in the infarcted myocardium was shown in part to be attenuation of apoptosis and senescence of cardiac fibroblasts by the activation of NRG1/ErbB/PI3K/Akt signaling (Shiraishi et al., 2022).

However, NRG1 has also been proposed to induce cardiomyocyte hypertrophy (De Keulenaer et al., 2019). In a cardiac magnetic resonance study, it was shown that rhNRG1-treated rats had significantly increased left ventricular wall thickness after MI and increased plasma N-terminal B-type natriuretic peptide precursor levels in a rhNRG1 dose-dependent manner (Zurek et al., 2020), confirming the detrimental effects of rhNRG1-induced hypertrophy and worsened cardiac function.

#### 3.4.2 NRG1 with left ventricular function

The NRG1/ErbB system is essential for cardiac development, is activated in the early stages of compensated HF, under conditions of myocardial stress, and decreases with disease progression and decompensation (Lemmens et al., 2007; Mendes-Ferreira et al., 2013). NRG1 administration improves cardiac dysfunction and reduces mortality in several models of LV (left ventricular) failure (Liu et al., 2006), and clinical trials have demonstrated the efficacy and safety of NRG1 in improving LV function in patients with HF (Gao et al., 2010; Jabbour et al., 2011a).

Abnormal Ca^2+^ handling in cardiac myocytes has been shown to affect excitation-contraction coupling and cellular function, leading to systolic dysfunction and arrhythmias (Johnson and Antoons, 2018). A recent study found that NRG1β increased Ca^2+^ current density in LV cardiomyocytes and promoted Ca^2+^ handling protein expression levels, which improved LV cardiac function in volume-overloaded HF rats (Wang et al., 2019). However, this experiment lacks direct evidence of an effect on calcium-handling function.

RhNRG1 is currently being tested in clinical trials as a new treatment for HF (Gao et al., 2010; Jabbour et al., 2011a). Early studies found that recombinant NRG1β improved myocardial contractility and diastolic capacity in rat models of chronic HF induced by ischemic, dilated, and viral cardiomyopathies (Liu et al., 2006). Similar improvements in cardiac function have been observed in dogs (Liu et al., 2006) and primates (Li et al., 2007). NRG1 attenuates the development of HF in several animal models, including diabetic cardiomyopathy (Liu et al., 2006; Odiete et al., 2013; Vandekerckhove et al., 2016). Advanced cardiomyopathy is an important cause of HF (Seidman and Seidman, 2001). Evidence suggests that weakening ErbB2 activity in the heart can directly contribute to cardiomyopathy and HF (Ozcelik et al., 2002). Therefore, ErbB2 holds promise as a potential target for clinical intervention in HF by NRG1. However, despite proposed explanations for the improvement in cardiomyocyte structure, contractility and proliferation of NRG1 (De Keulenaer et al., 2019), few studies have reported its effects on cardiac output and overall longitudinal strain, as well as LVEF and LV volume (Zurek et al., 2020), making it difficult to fully explain the benefits of NRG1 on left heart function.

### 3.5 NRG1 and cardiotoxicity

NRG1β is expressed in the cardiac microvascular endothelium, and promotes the growth and survival of cardiac myocytes in culture through the activation of ErbB2 and ErbB4 receptor tyrosine kinases (Fukazawa et al., 2003). On the one hand, NRG1*β* could reduce circulating levels of proinflammatory cytokines in rats with sepsis, adjust diaphragmatic proinflammatory cytokine level, mitigate diaphragmatic oxidative injury, and lessen diaphragm cell apoptosis, improving the function of the diaphragm and playing a role in the protection of the diaphragm by activating the signalling of PI3K/Akt (Liu et al., 2020). On the other hand, expression of NRG1 and recombinant NRG1β protects myocytes from anthracycline and β-adrenergic receptor-induced cell injury and death (Zhao et al., 1998; Sawyer et al., 2002; Fukazawa et al., 2003; Kuramochi et al., 2004b).

The pro-survival effect of NRG1 occurs through activation of ErbB2/4 in myocytes and downstream signaling in the PI3K/Akt pathway. Direct evidence for the cardiotoxic effects of NRG1 against anthracyclines is the protective activity of NRG1 against adriamycin-mediated myofibrillar disorders and prevention of troponin toxic degradation in cultured cardiomyocytes (Sawyer et al., 2002; Bian et al., 2009). NRG1 cardioprotective activity was also inferred *in vivo* by adriamycin-aggravated systolic dysfunction in heterozygous NRG1 mutant mice and exacerbated chamber dilation in ErbB4 knockout mice specific to the ventricles (Liu et al., 2005; Vasti et al., 2012). Therefore, expression of NRG1/ErbB not only promotes the proliferation of cardiomyocytes and reduces myocardial damage, but also reduces anthraquinone cardiotoxicity, which is undoubtedly beneficial for populations that require anthraquinone applications.

### 3.6 NRG1 and arrhythmia

NRG1 regulates myocardial function and sympathetic vagal homeostasis and is dynamically involved in the hemodynamic homeostasis of the cardiovascular system. NRG1 desensitizes the myocardium to the positive inotropic effects of isoprenaline through activation of eNOS, providing regulatory feedback on the autonomic imbalance present in acute cardiac stress and chronic HF (Zhao et al., 1999; Lemmens et al., 2004). Cardiomyocytes lacking NRG1 signaling are unable to adequately balance β-adrenergic activation by inhibiting parasympathetic activity (Okoshi et al., 2004; Brero et al., 2010).

Conditional ErbB2 mutant mice also exhibit prolonged ventricular repolarization time (increased QTc) and tachycardia (Ozcelik et al., 2002), which are not normally associated with dilated cardiomyopathy in humans. NRG1 has been reported to affect ventricular contractility and heart rate in perfused rat hearts as well as currents in K^+^-isolated sinus node myocytes (Wu et al., 2000). Changes in K^+^ or Na^+^ channel activity in humans and mice result in increased QTc and tip twisting arrhythmias (Keating and Sanguinetti, 2001). ErbB receptors classically activate various signaling cascades and affect K^+^ channels, non-selective cation channels and G protein-coupled receptors, all of which affect cardiac function (Wischmeyer et al., 1998; Prenzel et al., 1999; Schaefer et al., 2000).

## 4 NRG1 as a potential therapeutic target for cardiac repair

NRG1 treatment improves volume overload (Wang et al., 2012), adriamycin-induced LV dysfunction (Bian et al., 2009), as well as ischemia (Cohen et al., 2014) and diabetic cardiomyopathy (Li et al., 2011). Studies using NRG1 for cardiovascular regeneration and repair have focused on exogenous administration, and *in vivo* and *in vitro* experiments have confirmed its clinical effectiveness, respectively, and its greater potential and clinical value in the treatment of cardiovascular disease.

### 4.1 RhNRG1 and engineered bivalent NRG1β

The previously research shown that treatment with rhNRG1 improves pulmonary arterial hypertension by decreasing pulmonary arterial remodelling and endothelial dysfunction, as well as by restoring right ventricular function (Mendes-Ferreira et al., 2016). And recent study demonstrated that rhNRG1 treatment can decrease right ventricular intrinsic diastolic stiffness, through the improvement of calcium handling and cardiac remodelling signalling (Adão et al., 2019). The conclusion in Australia trial showed that short-term administration of rhNRG-1 results in acute and sustained improvement in cardiac function but it is an open-label study without a placebo group. In phase II clinical trials of rhNRG1 in the treatment of patients with chronic HF (Gao et al., 2010; Jabbour et al., 2011a) of China, Short-term administration of rhNRG-1 (0.6 μg/kg) in chronic HF patients could result in sustained improvement of cardiac pumping and inhibition or reversal of ventricular remodeling even 3 months after treatment, which is safe and well tolerated. RhNRG1 is emerging as a promising therapeutic option for cardiovascular disease and cardiac dysfunction.

RhNRG1 stimulates the proliferation of embryonic/fetal/neonatal cardiomyocytes, hypertrophic growth, sarcoma formation, and survival in isolation (Zhao et al., 1998; Lai et al., 2010). Although ErbB2 and ErbB4 play an important role in NRG1-promoted cardiac repair, overexpression of ErbB2 receptor subunits can promote uncontrolled cancer growth (Wadugu and Kühn, 2012), which would be the greatest limitation of clinical interventions to achieve myocardial regeneration with ErbB2. Previous studies have shown that ErbB3 play an adaptive role in overcoming the heart pressure overload (Yin et al., 2021) and the human protein database shows that the expression of ErbB3 is similar between immune cells and heart fibroblasts. Therefore, its therapeutic effect on heart disease cannot be ignored and is expected to be a potential target for overcoming rhNRG1 defects. Based on this theory, Lee et al. (Jay et al., 2013) designed engineered bivalent NRG1β, an effect mediated by receptor biasing toward ErbB3 homotypic interactions uncommonly formed by native NRG1β, resulting in some cases in decreased migration, inhibited proliferation, and increased apoptosis (Jay et al., 2011). Engineered bivalent NRG1β exhibits reduced tumor potential compared to NRG1 and still retains its cardioprotective properties (Jay et al., 2011; Jay et al., 2013).

### 4.2 NRG1 gene transduction

Gene fusions are heterozygous genes generated by structural DNA rearrangements, including translocations and insertions, transcriptional passages or splicing (Latysheva and Babu, 2016), resulting in dysregulated activity. The mechanism involving NRG1 gene fusions that induce cancer is due to ErbB-mediated pathway activation (Laskin et al., 2020) leading to aberrant cell proliferation. NRG1 fusions are enriched in aggressive mucinous adenocarcinoma of the lung, but have a low incidence in multiple tumor types (Nakaoku et al., 2014; Jones et al., 2017; Heining et al., 2018; Trombetta et al., 2018). Gene based therapies using gene delivery systems (eg., viral and nonviral vectors) to regulate gene expression at the cellular level can treat post-infarction pathological changes (Cao et al., 2019). In an experimental study in which a lentivirus carrying the human NRG1 gene was injected into the infarcted myocardium of rats, NRG1 gene transduction of the established a stable expression system in the infarcted heart and further activated the PI3K/Akt/eNOS pathway to promote neovascularization and prevent apoptosis (Xiao et al., 2012). In general, gene-based NRG1 therapy helps to reduce post-infarction cardiomyocyte loss, promote neoangiogenesis, and improve cardiac function. Furthermore, two studies have confirmed that endogenous up-regulation of NRG1/ErbB2 signaling and the promotion of NRG1 expression can also be achieved through exercise training (Cai et al., 2016; Cai et al., 2018), suggesting that there is great room for the development of methods to up-regulate NRG1 expression.

### 4.3 NRG1 and other growth factors

VEGF (vascular endothelial growth factor) and angiopoietin (Ang)-1 may regulate myocardial angiogenesis and survival via the NRG1/ErbB signaling pathway. VEGF or Ang-1 can significantly promote NRG1 expression and secretion in human cardiac microvascular endothelial cells (Wu et al., 2018). In turn, NRG1 treatment also increased significantly the expression of VEGF and Ang-1 in human coronary artery smooth muscle cells (Gui et al., 2018). Qiliqiangxin could attenuate anoxia-induced injuries in cardiac microvascular endothelial cells via NRG1/ErbB signalling which involves repairing damaged myocardial endothelial cells by affecting the expression and secretion levels of NRG1 and VEGF (Wang et al., 2017b).

These findings indicated NRG1 could increase the myocardial angiogenesis, probably via the direct effects of NRG1 and via the increasing expression of VEGF and Ang1 which provide a reliable basis for NRG combined with VEGF and other growth factors for cardiac repair. Furthermore, Lemmens et al. reported that mechanical strain increases endothelial NRG1 synthesis and release, but ang-II and adrenergic agonists decrease endothelial NRG1 synthesis and release (Lemmens et al., 2006; Lemmens et al., 2007). The mechanisms of the inverse relationship of Ang-2 on NRG1 are not clear and further research is still needed.

## 5 Concluding remarks and future directions

Successful cardiac repair requires three key phenomena: myocardial cell replenishment, removal of interstitial fibrosis, and hematologic reconstitution of the regenerating myocardium. Current research has focused on the direction of post-infarction myocardial proliferation, with less research in the cardiac microenvironment. Promotion of cardiac repair through the intervention of cardiac growth factor NRG1 has become a hot topic of current research. NRG1 has therapeutic effects on many forms of heart disease by directly acting on cardiomyocytes, endothelial cells, macrophages, and fibroblasts to promote cell proliferation, anti-apoptosis, anti-inflammatory and antioxidant effects, and regulate myocardial energy metabolism.

Biomaterials have emerged as innovative scaffolds for the delivery of both cells and proteins in tissue engineering applications. The combination of NRG-encapsulating scaffolds with cells capable of inducing cardiac regeneration could represent an ambitious and promising therapeutic strategy for the repair of diseased or damaged myocardial tissue (Simón-Yarza et al., 2015). Because nanoscale phenomena play an important role in cell signal transduction, enzyme action and cell cycle (Li et al., 2018), and shows excellent performance in the field of targeted drug therapy and the development of biomaterials, nanotechnology will hopefully be used in conjunction with NRG1 to repair the heart. Other emerging technology, such as DNA nano-threads (Baig et al., 2020), it can achieve deliver targeted drug through circular DNA scaffolding for the potential applications. These emerging technologies can accelerate the development of NRG1 therapy for cardiovascular diseases.

The mechanisms by which NRG/ErbB signaling is cardioprotective have been elucidated. The results of promising clinical trials of two different forms of recombinant NRG1 in systolic heart failure support further investigation of this biologic therapy. However, NRG1/ErbB still needs a lot of experiments to explore safe and feasible methods because it is hampered by the low efficiency of improving cardiac regeneration and side effects. And current experimental models are mainly limited to nonhuman animal models and to *in vitro* cellular models, it is difficult to completely replicate the real myocardial microenvironment of human. But it provides a direction for future research on cardiac repair in cardiovascular diseases.

## Glossary

ADAM: a disintegrin and metalloproteinase
AREG: amphiregulin
ATG5: autophagy related 5
AMI: acute myocardial infarction
ACAT-1: acyltransferase-1
COX-2: cyclooxygenase 2
CRD: cystein-rich domain
cMLCK: cardiac myosin light chain kinase
ECD: extracellular structural domain
EGF: epidermal growth factor
eNOS: Endothelial nitric oxide synthase
ERK: extracellular signal-regulated kinase
EPDCs: epicardial derived cells
ECM: extracellular matrix
FGF2: fibroblast growth factor 2
GSK3β: glycogen synthase kinase 3β
GLUT4: glomerular glucose transporter 4
GPx1: glutathione peroxidase 1
HF: heart failure
HIF-1α: hypoxia-inducible factor-1α
IR: ischemia/reperfusion
ICD: intracellular structural domain
IGF: insulin-like growth factor
LATSs: large tumor suppressor kinases
LVEF: left ventricular ejection fraction
LV: left ventricular
LDL: low-density lipoprotein
MI: myocardial infarction
MMP: matrix metalloproteinases
MSTs: Mammalian sterile 20-like kinases
MOBs: mps one binder kinase activator proteins
mTOR: rapamycin
MEK: mitogen-extracellular activated protein kinase
MAPK: adenosine monophosphate-activated protein kinase
NRG-1: Neuregulin-1
PI3K: phosphatidylinositol-3-kinase
Pik3cb: phosphatidylinositol-4,5-bisphosphate 3-kinase catalytic subunit beta
Akt-AS160: Akt substrate of 160 kDa (AS160)
RASSFs: ras associated domain family
PDK1: phosphatidylinositol-dependent kinase 1
rhNRG1: recombinant human NRG1
ROS: reactive oxygen species
RIPC: remote ischemic preconditioning
SAV1: salvador homolog 1
SASPs: senescence-associated secretory phenotypes
SIPS: stress-induced premature senescence
TAZ: transcriptional coactivator with PDZ-binding motif
TEAD: transcriptional enhanced associate domain
Trx2: thioredoxin-2
VEGFA: vascular endothelial growth factor A
VSMCs: vascular smooth muscle cells
YAP: yes-associated protein
VEGF: vascular endothelial growth factor

## References

[B1] Abais J. M. Xia M. Zhang Y. Boini K. M. Li P. L. (2015). Redox regulation of NLRP3 inflammasomes: ROS as trigger or effector? Antioxid. Redox Signal. 22 (13), 1111–1129. 10.1089/ars.2014.5994 25330206 PMC4403231

[B2] Acharya A. Baek S. T. Huang G. Eskiocak B. Goetsch S. Sung C. Y. (2012). The bHLH transcription factor Tcf21 is required for lineage-specific EMT of cardiac fibroblast progenitors. Development 139 (12), 2139–2149. 10.1242/dev.079970 22573622 PMC3357908

[B3] Adão R. Mendes-Ferreira P. Maia-Rocha C. Santos-Ribeiro D. Rodrigues P. G. Vidal-Meireles A. (2019). Neuregulin-1 attenuates right ventricular diastolic stiffness in experimental pulmonary hypertension. Clin. Exp. Pharmacol. Physiol. 46 (3), 255–265. 10.1111/1440-1681.13043 30339273

[B4] Aharonov A. Shakked A. Umansky K. B. Savidor A. Genzelinakh A. Kain D. (2020). ERBB2 drives YAP activation and EMT-like processes during cardiac regeneration. Nat. Cell Biol. 22 (11), 1346–1356. 10.1038/s41556-020-00588-4 33046882

[B5] Amin D. N. Hida K. Bielenberg D. R. Klagsbrun M. (2006). Tumor endothelial cells express epidermal growth factor receptor (EGFR) but not ErbB3 and are responsive to EGF and to EGFR kinase inhibitors. Cancer Res. 66 (4), 2173–2180. 10.1158/0008-5472.CAN-05-3387 16489018

[B6] An T. Huang Y. Zhou Q. Wei B. Q. Zhang R. C. Yin S. J. (2013). Neuregulin-1 attenuates doxorubicin-induced autophagy in neonatal rat cardiomyocytes. J. Cardiovasc. Pharmacol. 62 (2), 130–137. 10.1097/FJC.0b013e318291c094 23519142

[B7] Arora H. Lavin A. C. Balkan W. Hare J. M. White I. A. (2021). Neuregulin-1, in a conducive milieu with Wnt/BMP/retinoic acid, prolongs the epicardial-mediated cardiac regeneration capacity of neonatal heart explants. J. Stem Cells Regen. Med. 17 (1), 18–27. 10.46582/jsrm.1701003 34434004 PMC8372415

[B8] Kajstura J. Gurusamy N. Ogórek B. Goichberg P. Clavo-Rondon C. Hosoda T. (2010). Myocyte turnover in the aging human heart [retracted in: Circ Res. 2019 Feb 15;124(4): e23]. Circ. Res. 107 (11), 1374–1386. 10.1161/CIRCRESAHA.110.231498 21088285

[B9] Baig M. Lai W. F. Ahsan A. Jabeen M. Farooq M. A. Mikrani R. (2020). Synthesis of ligand functionalized ErbB-3 targeted novel DNA nano-threads loaded with the low dose of doxorubicin for efficient *in vitro* evaluation of the resistant anti-cancer activity. Pharm. Res. 37 (4), 75. 10.1007/s11095-020-02803-1 32232574

[B10] Bao J. Lin H. Ouyang Y. Lei D. Osman A. Kim T. W. (2004). Activity-dependent transcription regulation of PSD-95 by neuregulin-1 and Eos. Nat. Neurosci. 7 (11), 1250–1258. 10.1038/nn1342 15494726

[B11] Bao J. Wolpowitz D. Role L. W. Talmage D. A. (2003). Back signaling by the Nrg-1 intracellular domain. J. Cell Biol. 161 (6), 1133–1141. 10.1083/jcb.200212085 12821646 PMC2172983

[B12] Belmonte F. Das S. Sysa-Shah P. Sivakumaran V. Stanley B. Guo X. (2015). ErbB2 overexpression upregulates antioxidant enzymes, reduces basal levels of reactive oxygen species, and protects against doxorubicin cardiotoxicity. Am. J. Physiol. Heart Circ. Physiol. 309 (8), H1271–H1280. 10.1152/ajpheart.00517.2014 26254336 PMC4666964

[B13] Bersell K. Arab S. Haring B. Kuhn B. (2009). Neuregulin1/ErbB4 signaling induces cardiomyocyte proliferation and repair of heart injury. Cell 138 (2), 257–270. 10.1016/j.cell.2009.04.060 19632177

[B14] Bian Y. Sun M. Silver M. Ho K. K. L. Marchionni M. A. Caggiano A. O. (2009). Neuregulin-1 attenuated doxorubicin-induced decrease in cardiac troponins. Am. J. Physiol. Heart Circ. Physiol. 297 (6), H1974–H1983. 10.1152/ajpheart.01010.2008 19801490 PMC2793128

[B15] Bouyain S. Longo P. A. Li S. Ferguson K. M. Leahy D. J. (2005). The extracellular region of ErbB4 adopts a tethered conformation in the absence of ligand. Proc. Natl. Acad. Sci. U. S. A. 102 (42), 15024–15029. 10.1073/pnas.0507591102 16203964 PMC1257738

[B16] Brero A. Ramella R. Fitou A. Dati C. Alloatti G. Gallo M. P. (2010). Neuregulin-1beta1 rapidly modulates nitric oxide synthesis and calcium handling in rat cardiomyocytes. Cardiovasc. Res. 88 (3), 443–452. 10.1093/cvr/cvq238 20634213

[B17] Buonanno A. Fischbach G. D. (2001). Neuregulin and ErbB receptor signaling pathways in the nervous system. Curr. Opin. Neurobiol. 11 (3), 287–296. 10.1016/s0959-4388(00)00210-5 11399426

[B18] Cai M. Wang Q. Liu Z. Jia D. Feng R. Tian Z. (2018). Effects of different types of exercise on skeletal muscle atrophy, antioxidant capacity and growth factors expression following myocardial infarction. Life Sci. 213, 40–49. 10.1016/j.lfs.2018.10.015 30312703

[B19] Cai M. X. Shi X. C. Chen T. Tan Z. N. Lin Q. Q. Du S. J. (2016). Exercise training activates neuregulin 1/ErbB signaling and promotes cardiac repair in a rat myocardial infarction model. Life Sci. 149, 1–9. 10.1016/j.lfs.2016.02.055 26892146

[B20] Caja L. Sancho P. Bertran E. Iglesias-Serret D. Gil J. Fabregat I. (2009). Overactivation of the MEK/ERK pathway in liver tumor cells confers resistance to TGF-{beta}-induced cell death through impairing up-regulation of the NADPH oxidase NOX4. Cancer Res. 69 (19), 7595–7602. 10.1158/0008-5472.CAN-09-1482 19773433

[B21] Campreciós G. Lorita J. Pardina E. Peinado-Onsurbe J. Soley M. Ramirez I. (2011). Expression, localization, and regulation of the neuregulin receptor ErbB3 in mouse heart. J. Cell. Physiol. 226 (2), 450–455. 10.1002/jcp.22354 20672328

[B22] Cao Y. Tan Y. F. Wong Y. S. Liew M. W. J. Venkatraman S. (2019). Recent advances in chitosan-based carriers for gene delivery. Mar. Drugs 17 (6), E381. 10.3390/md17060381 31242678 PMC6627531

[B23] Carraway K. L. 3rd Cantley L. C. (1994). A neu acquaintance for erbB3 and erbB4: A role for receptor heterodimerization in growth signaling. Cell 78 (1), 5–8. 10.1016/0092-8674(94)90564-9 8033211

[B24] Chou C. F. Ozaki M. (2010). *In silico* analysis of neuregulin 1 evolution in vertebrates. Biosci. Rep. 30 (4), 267–275. 10.1042/BSR20090097 19681757

[B25] Clement C. M. Thomas L. K. Mou Y. Croslan D. R. Gibbons G. H. Ford B. D. (2007). Neuregulin-1 attenuates neointimal formation following vascular injury and inhibits the proliferation of vascular smooth muscle cells. J. Vasc. Res. 44 (4), 303–312. 10.1159/000101776 17438359

[B26] Cohen J. E. Purcell B. P. MacArthur J. W. Mu A. Shudo Y. Patel J. B. (2014). A bioengineered hydrogel system enables targeted and sustained intramyocardial delivery of neuregulin, activating the cardiomyocyte cell cycle and enhancing ventricular function in a murine model of ischemic cardiomyopathy. Circ. Heart Fail. 7 (4), 619–626. 10.1161/CIRCHEARTFAILURE.113.001273 24902740 PMC4157671

[B27] Colliva A. Braga L. Giacca M. Zacchigna S. (2020). Endothelial cell-cardiomyocyte crosstalk in heart development and disease. J. Physiol. 598 (14), 2923–2939. 10.1113/JP276758 30816576 PMC7496632

[B28] Correia-Melo C. Hewitt G. Passos J. F. (2014). Telomeres, oxidative stress and inflammatory factors: Partners in cellular senescence? Longev. Heal. 3 (1), 1. 10.1186/2046-2395-3-1 PMC3922784 24472138

[B29] Cote G. M. Miller T. A. Lebrasseur N. K. Kuramochi Y. Sawyer D. B. (2005). Neuregulin-1alpha and beta isoform expression in cardiac microvascular endothelial cells and function in cardiac myocytes *in vitro* . Exp. Cell Res. 311 (1), 135–146. 10.1016/j.yexcr.2005.08.017 16185687

[B30] Crone S. A. Zhao Y. Y. Fan L. Gu Y. Minamisawa S. Liu Y. (2002). ErbB2 is essential in the prevention of dilated cardiomyopathy. Nat. Med. 8 (5), 459–465. 10.1038/nm0502-459 11984589

[B31] D'Uva G. Aharonov A. Lauriola M. Kain D. Yahalom-Ronen Y. Carvalho S. (2015). ERBB2 triggers mammalian heart regeneration by promoting cardiomyocyte dedifferentiation and proliferation. Nat. Cell Biol. 17 (5), 627–638. 10.1038/ncb3149 25848746

[B32] De Keulenaer G. W. Feyen E. Dugaucquier L. Shakeri H. Shchendrygina A. Belenkov Y. N. (2019). Mechanisms of the multitasking endothelial protein NRG-1 as a compensatory factor during chronic heart failure. Circ. Heart Fail. 12 (10), e006288. 10.1161/CIRCHEARTFAILURE.119.006288 31607147

[B33] Del Re D. P. Amgalan D. Linkermann A. Liu Q. Kitsis R. N. (2019). Fundamental mechanisms of regulated cell death and implications for heart disease. Physiol. Rev. 99 (4), 1765–1817. 10.1152/physrev.00022.2018 31364924 PMC6890986

[B34] Dey A. Varelas X. Guan K. L. (2020). Targeting the Hippo pathway in cancer, fibrosis, wound healing and regenerative medicine. Nat. Rev. Drug Discov. 19 (7), 480–494. 10.1038/s41573-020-0070-z 32555376 PMC7880238

[B35] Ding Z. Dai C. Zhong L. Liu R. Gao W. Zhang H. (2021). Neuregulin-1 converts reactive astrocytes toward oligodendrocyte lineage cells via upregulating the PI3K-AKT-mTOR pathway to repair spinal cord injury. Biomed. Pharmacother. 134, 111168. 10.1016/j.biopha.2020.111168 33395598

[B36] Du Y. Yang H. Xu Y. Cang X. Luo C. Mao Y. (2012). Conformational transition and energy landscape of ErbB4 activated by neuregulin1β: One microsecond molecular dynamics simulations. J. Am. Chem. Soc. 134 (15), 6720–6731. 10.1021/ja211941d 22316159

[B37] Dugaucquier L. Feyen E. Mateiu L. Bruyns T. A. M. De Keulenaer G. W. Segers V. F. M. (2020). The role of endothelial autocrine NRG1/ERBB4 signaling in cardiac remodeling. Am. J. Physiol. Heart Circ. Physiol. 319 (2), H443–H455. 10.1152/ajpheart.00176.2020 32618511

[B38] Erkens R. Totzeck M. Brum A. Duse D. Botker H. E. Rassaf T. (2021). Endothelium-dependent remote signaling in ischemia and reperfusion: Alterations in the cardiometabolic continuum. Free Radic. Biol. Med. 165, 265–281. 10.1016/j.freeradbiomed.2021.01.040 33497796

[B39] Falls D. L. (2003). Neuregulins: Functions, forms, and signaling strategies. Exp. Cell Res. 284 (1), 14–30. 10.1016/s0014-4827(02)00102-7 12648463

[B40] Falls D. L. Rosen K. M. Corfas G. Lane W. S. Fischbach G. D. (1993). ARIA, a protein that stimulates acetylcholine receptor synthesis, is a member of the neu ligand family. Cell 72 (5), 801–815. 10.1016/0092-8674(93)90407-h 8453670

[B41] Fang S. J. Li P. Y. Wang C. M. Xin Y. Lu W. W. Zhang X. X. (2017). Inhibition of endoplasmic reticulum stress by neuregulin-1 protects against myocardial ischemia/reperfusion injury. Peptides 88, 196–207. 10.1016/j.peptides.2016.12.009 27993557

[B42] Fang S. J. Wu X. S. Han Z. H. Zhang X. X. Wang C. M. Li X. Y. (2010). Neuregulin-1 preconditioning protects the heart against ischemia/reperfusion injury through a PI3K/Akt-dependent mechanism. Chin. Med. J. 123 (24), 3597–3604. 22166638

[B43] Fernandez-Cuesta L. Plenker D. Osada H. Sun R. Menon R. Leenders F. (2014). CD74-NRG1 fusions in lung adenocarcinoma. Cancer Discov. 4 (4), 415–422. 10.1158/2159-8290.CD-13-0633 24469108

[B44] Fukazawa R. Miller T. A. Kuramochi Y. Frantz S. Kim Y. D. Marchionni M. A. (2003). Neuregulin-1 protects ventricular myocytes from anthracycline-induced apoptosis via erbB4-dependent activation of PI3-kinase/Akt. J. Mol. Cell. Cardiol. 35 (12), 1473–1479. 10.1016/j.yjmcc.2003.09.012 14654373

[B45] Galindo C. L. Ryzhov S. Sawyer D. B. (2014). Neuregulin as a heart failure therapy and mediator of reverse remodeling. Curr. Heart Fail. Rep. 11 (1), 40–49. 10.1007/s11897-013-0176-2 24234399 PMC3975684

[B46] Gao R. Zhang J. Cheng L. Wu X. Dong W. Yang X. (2010). A Phase II, randomized, double-blind, multicenter, based on standard therapy, placebo-controlled study of the efficacy and safety of recombinant human neuregulin-1 in patients with chronic heart failure. J. Am. Coll. Cardiol. 55 (18), 1907–1914. 10.1016/j.jacc.2009.12.044 20430261

[B47] Garratt A. N. Britsch S. Birchmeier C. (2000). Neuregulin, a factor with many functions in the life of a schwann cell. Bioessays 22 (11), 987–996. 10.1002/1521-1878(200011)22:11<987::AID-BIES5>3.0.CO;2-5 11056475

[B48] Gassmann M. CasagrandaF. Orioli D. Simon H. Lai C. Klein R. (1995). Aberrant neural and cardiac development in mice lacking the ErbB4 neuregulin receptor. Nature 378 (6555), 390–394. 10.1038/378390a0 7477376

[B49] Gemberling M. Karra R. Dickson A. L. Poss K. D. (2015). Nrg1 is an injury-induced cardiomyocyte mitogen for the endogenous heart regeneration program in zebrafish. Elife 4, e05871. 10.7554/eLife.05871 PMC4379493 25830562

[B50] Gibb A. A. Lazaropoulos M. P. Elrod J. W. (2020). Myofibroblasts and fibrosis: Mitochondrial and metabolic control of cellular differentiation. Circ. Res. 127 (3), 427–447. 10.1161/CIRCRESAHA.120.316958 32673537 PMC7982967

[B51] Gorenne I. Kavurma M. Scott S. Bennett M. (2006). Vascular smooth muscle cell senescence in atherosclerosis. Cardiovasc. Res. 72 (1), 9–17. 10.1016/j.cardiores.2006.06.004 16824498

[B52] Griffiths H. R. Gao D. Pararasa C. (2017). Redox regulation in metabolic programming and inflammation. Redox Biol. 12 (C), 50–57. 10.1016/j.redox.2017.01.023 28212523 PMC5312548

[B53] Grimm S. Leder P. (1997). An apoptosis-inducing isoform of neu differentiation factor (NDF) identified using a novel screen for dominant, apoptosis-inducing genes. J. Exp. Med. 185 (6), 1137–1142. 10.1084/jem.185.6.1137 9091587 PMC2196240

[B54] Grimm S. Weinstein E. J. Krane I. M. Leder P. (1998). Neu differentiation factor (NDF), a dominant oncogene, causes apoptosis *in vitro* and *in vivo* . J. Exp. Med. 188 (8), 1535–1539. 10.1084/jem.188.8.1535 9782131 PMC2213420

[B55] Grines C. L. Watkins M. W. Helmer G. Penny W. Brinker J. Marmur J. D. (2002). Angiogenic Gene Therapy (AGENT) trial in patients with stable angina pectoris. Circulation 105 (11), 1291–1297. 10.1161/hc1102.105595 11901038

[B56] Gu X. Liu X. Xu D. Li X. Yan M. Qi Y. (2010). Cardiac functional improvement in rats with myocardial infarction by up-regulating cardiac myosin light chain kinase with neuregulin. Cardiovasc. Res. 88 (2), 334–343. 10.1093/cvr/cvq223 20615916

[B57] Gui C. Zeng Z. Y. Chen Q. Luo Y. W. Li L. Chen L. L. (2018). Neuregulin-1 promotes myocardial angiogenesis in the rat model of diabetic cardiomyopathy. Cell. Physiol. biochem. 46 (6), 2325–2334. 10.1159/000489622 29742506

[B58] Guo Y. F. Zhang X. x. Liu Y. Duan H. y. Jie B. z. Wu X. s. (2012). Neuregulin-1 attenuates mitochondrial dysfunction in a rat model of heart failure. Chin. Med. J. 125 (5), 807–814. 22490579

[B59] Gupte M. Lal H. Ahmad F. Sawyer D. B. Hill M. F. (2017). Chronic neuregulin-1β treatment mitigates the progression of postmyocardial infarction heart failure in the setting of type 1 diabetes mellitus by suppressing myocardial apoptosis, fibrosis, and key oxidant-producing enzymes. J. Card. Fail. 23 (12), 887–899. 10.1016/j.cardfail.2017.08.456 28870731 PMC5716474

[B60] Haller P. M. Goncalves I. F. Acar E. Jager B. Pilz P. M. Wojta J. (2022). Relationship between plasma Neuregulin-1 and cardiac function in patients with ST-elevation myocardial infarction. Rev. Cardiovasc. Med. 23 (2), 63. 10.31083/j.rcm2302063 35229554

[B61] Harvey K. F. Zhang X. Thomas D. M. (2013). The Hippo pathway and human cancer. Nat. Rev. Cancer 13 (4), 246–257. 10.1038/nrc3458 23467301

[B62] Haskins J. W. Nguyen D. X. Stern D. F. (2014). Neuregulin 1-activated ERBB4 interacts with YAP to induce Hippo pathway target genes and promote cell migration. Sci. Signal. 7 (355), ra116. 10.1126/scisignal.2005770 25492965 PMC4648367

[B63] Heallen T. Morikawa Y. Leach J. Tao G. Willerson J. T. Johnson R. L. (2013). Hippo signaling impedes adult heart regeneration. Development 140 (23), 4683–4690. 10.1242/dev.102798 24255096 PMC3833428

[B64] Heallen T. Zhang M. Wang J. Bonilla-Claudio M. Klysik E. Johnson R. L. (2011). Hippo pathway inhibits Wnt signaling to restrain cardiomyocyte proliferation and heart size. Science 332 (6028), 458–461. 10.1126/science.1199010 21512031 PMC3133743

[B65] Heallen T. Zhang M. Wang J. Bonilla-Claudio M. Klysik E. Johnson R. L. (2011). Hippo pathway inhibits Wnt signaling to restrain cardiomyocyte proliferation and heart size. Science 332 (6028), 458–461. 10.1126/science.1199010 21512031 PMC3133743

[B66] Hedhli N. Dobrucki L. W. Kalinowski A. Zhuang Z. W. Wu X. Russell R. R. (2012). Endothelial-derived neuregulin is an important mediator of ischaemia-induced angiogenesis and arteriogenesis. Cardiovasc. Res. 93 (3), 516–524. 10.1093/cvr/cvr352 22200588 PMC3282578

[B67] Hedhli N. Huang Q. Kalinowski A. Palmeri M. Hu X. Russell R. R. (2011). Endothelium-derived neuregulin protects the heart against ischemic injury. Circulation 123 (20), 2254–2262. 10.1161/CIRCULATIONAHA.110.991125 21555713 PMC3104502

[B68] Hedman M. Hartikainen J. Syvanne M. Stjernvall J. Hedman A. Kivela A. (2003). Safety and feasibility of catheter-based local intracoronary vascular endothelial growth factor gene transfer in the prevention of postangioplasty and in-stent restenosis and in the treatment of chronic myocardial ischemia: Phase II results of the kuopio angiogenesis trial (KAT). Circulation 107 (21), 2677–2683. 10.1161/01.CIR.0000070540.80780.92 12742981

[B69] Heining C. Horak P. Uhrig S. Codo P. L. Klink B. Hutter B. (2018). NRG1 fusions in KRAS wild-type pancreatic cancer. Cancer Discov. 8 (9), 1087–1095. 10.1158/2159-8290.CD-18-0036 29802158

[B70] Henry T. D. Annex B. H. McKendall G. R. Azrin M. A. Lopez J. J. Giordano F. J. (2003). The VIVA trial: Vascular endothelial growth factor in Ischemia for Vascular Angiogenesis. Circulation 107 (10), 1359–1365. 10.1161/01.cir.0000061911.47710.8a 12642354

[B71] Herget G. W. NeuburgerM. Plagwitz R. Adler C. P. (1997). DNA content, ploidy level and number of nuclei in the human heart after myocardial infarction. Cardiovasc. Res. 36 (1), 45–51. 10.1016/s0008-6363(97)00140-5 9415271

[B72] Hintsanen M. Elovainio M. Puttonen S. Kivimaki M. Raitakari O. T. Lehtimaki T. (2007). Neuregulin-1 genotype moderates the association between job strain and early atherosclerosis in young men. Ann. Behav. Med. 33 (2), 148–155. 10.1007/BF02879896 17447867

[B73] Holmes W. E. Sliwkowski M. X. Akita R. W. Henzel W. J. Lee J. Park J. W. (1992). Identification of heregulin, a specific activator of p185erbB2. Science 256 (5060), 1205–1210. 10.1126/science.256.5060.1205 1350381

[B74] Hong X. Oh N. Wang K. Neumeyer J. Lee C. N. Lin R. Z. (2021). Human endothelial colony-forming cells provide trophic support for pluripotent stem cell-derived cardiomyocytes via distinctively high expression of neuregulin-1. Angiogenesis 24 (2), 327–344. 10.1007/s10456-020-09765-3 33454888 PMC8337094

[B75] Honkoop H. de Bakker D. E. Aharonov A. Kruse F. Shakked A. Nguyen P. D. (2019). Single-cell analysis uncovers that metabolic reprogramming by ErbB2 signaling is essential for cardiomyocyte proliferation in the regenerating heart. Elife 8, e50163. 10.7554/eLife.50163 31868166 PMC7000220

[B76] Horiuchi K. Zhou H. M. Kelly K. Manova K. Blobel C. P. (2005). Evaluation of the contributions of ADAMs 9, 12, 15, 17, and 19 to heart development and ectodomain shedding of neuregulins beta1 and beta2. Dev. Biol. 283 (2), 459–471. 10.1016/j.ydbio.2005.05.004 15936750

[B77] Hotham W. E. Henson F. M. D. (2020). The use of large animals to facilitate the process of MSC going from laboratory to patient-'bench to bedside. Cell Biol. Toxicol. 36 (2), 103–114. 10.1007/s10565-020-09521-9 32206986 PMC7196082

[B78] Huang Q. Zhang J. Liang L. Lan Z. Huo T. Li S. (2015). The significance of neuregulin-1/ErbB expression in autogenous vein grafts in a diabetic rat model. J. Cardiovasc. Pharmacol. 66 (3), 300–306. 10.1097/FJC.0000000000000279 25978692

[B79] Huang Q. Zhou H. J. Zhang H. Huang Y. Hinojosa-Kirschenbaum F. Fan P. (2015). Thioredoxin-2 inhibits mitochondrial reactive oxygen species generation and apoptosis stress kinase-1 activity to maintain cardiac function. Circulation 131 (12), 1082–1097. 10.1161/CIRCULATIONAHA.114.012725 25628390 PMC4374031

[B80] Ikenishi A. Okayama H. Iwamoto N. Yoshitome S. Tane S. Nakamura K. (2012). Cell cycle regulation in mouse heart during embryonic and postnatal stages. Dev. Growth Differ. 54 (8), 731–738. 10.1111/j.1440-169X.2012.01373.x 22957921

[B81] Jabbour A. Gao L. Kwan J. Watson A. Sun L. Qiu M. R. (2011). A recombinant human neuregulin-1 peptide improves preservation of the rodent heart after prolonged hypothermic storage. Transplantation 91 (9), 961–967. 10.1097/TP.0b013e3182115b4b 21364498

[B82] Jabbour A. Hayward C. S. Keogh A. M. Kotlyar E. McCrohon J. A. England J. F. (2011). Parenteral administration of recombinant human neuregulin-1 to patients with stable chronic heart failure produces favourable acute and chronic haemodynamic responses. Eur. J. Heart Fail. 13 (1), 83–92. 10.1093/eurjhf/hfq152 20810473

[B83] Janse van Rensburg H. J. Azad T. Ling M. Hao Y. Snetsinger B. Khanal P. (2018). The Hippo pathway component TAZ promotes immune evasion in human cancer through PD-L1. Cancer Res. 78 (6), 1457–1470. 10.1158/0008-5472.CAN-17-3139 29339539

[B84] Jay S. M. Kurtagic E. Alvarez L. M. de Picciotto S. Sanchez E. Hawkins J. F. (2011). Engineered bivalent ligands to bias ErbB receptor-mediated signaling and phenotypes. J. Biol. Chem. 286 (31), 27729–27740. 10.1074/jbc.M111.221093 21622572 PMC3149363

[B85] Jay S. M. Murthy A. C. Hawkins J. F. Wortzel J. R. Steinhauser M. L. Alvarez L. M. (2013). An engineered bivalent neuregulin protects against doxorubicin-induced cardiotoxicity with reduced proneoplastic potential. Circulation 128 (2), 152–161. 10.1161/CIRCULATIONAHA.113.002203 23757312 PMC3753575

[B86] Jiang K. Tu Z. Chen K. Xu Y. Chen F. Xu S. (2022). Gasdermin D inhibition confers antineutrophil-mediated cardioprotection in acute myocardial infarction. J. Clin. Invest. 132 (1), e151268. 10.1172/JCI151268 34752417 PMC8718151

[B87] Jiao L. Wang M. C. Yang Y. A. Chen E. Q. Xu H. T. Wu K. Y. (2008). Norepinephrine reversibly regulates the proliferation and phenotypic transformation of vascular smooth muscle cells. Exp. Mol. Pathol. 85 (3), 196–200. 10.1016/j.yexmp.2008.09.007 18976651

[B88] Johnson D. M. Antoons G. (2018). Arrhythmogenic mechanisms in heart failure: Linking β-adrenergic stimulation, stretch, and calcium. Front. Physiol. 9, 1453. 10.3389/fphys.2018.01453 30374311 PMC6196916

[B89] Jones J. T. Ballinger M. D. Pisacane P. I. Lofgren J. A. Fitzpatrick V. D. Fairbrother W. J. (1998). Binding interaction of the heregulinbeta egf domain with ErbB3 and ErbB4 receptors assessed by alanine scanning mutagenesis. J. Biol. Chem. 273 (19), 11667–11674. 10.1074/jbc.273.19.11667 9565587

[B90] Jones M. R. Lim H. Shen Y. PlEasancE E. Ch'ng C. Reisle C. (2017). Successful targeting of the NRG1 pathway indicates novel treatment strategy for metastatic cancer. Ann. Oncol. 28 (12), 3092–3097. 10.1093/annonc/mdx523 28950338

[B91] Karar J. Maity A. (2011). PI3K/AKT/mTOR pathway in angiogenesis. Front. Mol. Neurosci. 4 (51), 51. 10.3389/fnmol.2011.00051 22144946 PMC3228996

[B92] Kataria H. Alizadeh A. Karimi-Abdolrezaee S. (2019). Neuregulin-1/ErbB network: An emerging modulator of nervous system injury and repair. Prog. Neurobiol. 180, 101643. 10.1016/j.pneurobio.2019.101643 31229498

[B93] Katz T. C. Singh M. K. Degenhardt K. Rivera-Feliciano J. Johnson R. L. Epstein J. A. (2012). Distinct compartments of the proepicardial organ give rise to coronary vascular endothelial cells. Dev. Cell 22 (3), 639–650. 10.1016/j.devcel.2012.01.012 22421048 PMC3306604

[B94] Kawaguchi M. Takahashi M. Hata T. Kashima Y. Usui F. Morimoto H. (2011). Inflammasome activation of cardiac fibroblasts is essential for myocardial ischemia/reperfusion injury. Circulation 123 (6), 594–604. 10.1161/CIRCULATIONAHA.110.982777 21282498

[B95] Keating M. T. Sanguinetti M. C. (2001). Molecular and cellular mechanisms of cardiac arrhythmias. Cell 104 (4), 569–580. 10.1016/s0092-8674(01)00243-4 11239413

[B96] Kramer R. BucayN. Kane D. J. Martin L. E. Tarpley J. E. Theill L. E. (1996). Neuregulins with an Ig-like domain are essential for mouse myocardial and neuronal development. Proc. Natl. Acad. Sci. U. S. A. 93 (10), 4833–4838. 10.1073/pnas.93.10.4833 8643489 PMC39365

[B97] Kumar A. Harris T. E. Keller S. R. Choi K. M. Magnuson M. A. Lawrence J. C. (2008). Muscle-specific deletion of rictor impairs insulin-stimulated glucose transport and enhances Basal glycogen synthase activity. Mol. Cell. Biol. 28 (1), 61–70. 10.1128/MCB.01405-07 17967879 PMC2223287

[B98] Kundumani-Sridharan V. Subramani J. Owens C. Das K. C. (2021). Nrg1β released in remote ischemic preconditioning improves myocardial perfusion and decreases ischemia/reperfusion injury via ErbB2-mediated rescue of endothelial nitric oxide synthase and abrogation of Trx2 autophagy. Arterioscler. Thromb. Vasc. Biol. 41 (8), 2293–2314. 10.1161/ATVBAHA.121.315957 34039018 PMC8288485

[B99] Kuramochi Y. Cote G. M. Guo X. Lebrasseur N. K. Cui L. Liao R. (2004). Cardiac endothelial cells regulate reactive oxygen species-induced cardiomyocyte apoptosis through neuregulin-1beta/erbB4 signaling. J. Biol. Chem. 279 (49), 51141–51147. 10.1074/jbc.M408662200 15385548

[B100] Kuramochi Y. Lim C. C. Guo X. Colucci W. S. Liao R. Sawyer D. B. (2004). Myocyte contractile activity modulates norepinephrine cytotoxicity and survival effects of neuregulin-1beta. Am. J. Physiol. Cell Physiol. 286 (2), C222–C229. 10.1152/ajpcell.00312.2003 14522821

[B101] Kurohara K. Komatsu K. Kurisaki T. Masuda A. Irie N. Asano M. (2004). Essential roles of Meltrin beta (ADAM19) in heart development. Dev. Biol. 267 (1), 14–28. 10.1016/j.ydbio.2003.10.021 14975714

[B102] Ky B. Kimmel S. E. Safa R. N. Putt M. E. Sweitzer N. K. Fang J. C. (2009). Neuregulin-1 beta is associated with disease severity and adverse outcomes in chronic heart failure. Circulation 120 (4), 310–317. 10.1161/CIRCULATIONAHA.109.856310 19597049 PMC2741393

[B103] Lai D. Liu X. Forrai A. Wolstein O. Michalicek J. Ahmed I. (2010). Neuregulin 1 sustains the gene regulatory network in both trabecular and nontrabecular myocardium. Circ. Res. 107 (6), 715–727. 10.1161/CIRCRESAHA.110.218693 20651287

[B104] Laplante M. Sabatini D. M. (2012). mTOR signaling in growth control and disease. Cell 149 (2), 274–293. 10.1016/j.cell.2012.03.017 22500797 PMC3331679

[B105] Laskin J. Liu S. V. Tolba K. Heining C. Schlenk R. F. Cheema P. (2020). NRG1 fusion-driven tumors: Biology, detection, and the therapeutic role of afatinib and other ErbB-targeting agents. Ann. Oncol. 31 (12), 1693–1703. 10.1016/j.annonc.2020.08.2335 32916265 PMC8911318

[B106] Latysheva N. S. Babu M. M. (2016). Discovering and understanding oncogenic gene fusions through data intensive computational approaches. Nucleic Acids Res. 44 (10), 4487–4503. 10.1093/nar/gkw282 27105842 PMC4889949

[B107] Lázár E. Sadek H. A. Bergmann O. (2017). Cardiomyocyte renewal in the human heart: Insights from the fall-out. Eur. Heart J. 38 (30), 2333–2342. 10.1093/eurheartj/ehx343 28810672 PMC5837331

[B108] Lee K. F. Simon H. CHen H. Bates B. Hung M. C. Hauser C. (1995). Requirement for neuregulin receptor erbB2 in neural and cardiac development. Nature 378 (6555), 394–398. 10.1038/378394a0 7477377

[B109] Leimeroth R. Lobsiger C. Lussi A. Taylor V. Suter U. Sommer L. (2002). Membrane-bound neuregulin1 type III actively promotes Schwann cell differentiation of multipotent Progenitor cells. Dev. Biol. 246 (2), 245–258. 10.1006/dbio.2002.0670 12051814

[B110] Lemmens K. Doggen K. De Keulenaer G. W. (2011). Activation of the neuregulin/ErbB system during physiological ventricular remodeling in pregnancy. Am. J. Physiol. Heart Circ. Physiol. 300 (3), H931–H942. 10.1152/ajpheart.00385.2010 21186272

[B111] Lemmens K. Doggen K. De Keulenaer G. W. (2007). Role of neuregulin-1/ErbB signaling in cardiovascular physiology and disease: Implications for therapy of heart failure. Circulation 116 (8), 954–960. 10.1161/CIRCULATIONAHA.107.690487 17709650

[B112] Lemmens K. Fransen P. Sys S. U. Brutsaert D. L. De Keulenaer G. W. (2004). Neuregulin-1 induces a negative inotropic effect in cardiac muscle: Role of nitric oxide synthase. Circulation 109 (3), 324–326. 10.1161/01.CIR.0000114521.88547.5E 14732742

[B113] Lemmens K. Segers V. F. M. Demolder M. De Keulenaer G. W. (2006). Role of neuregulin-1/ErbB2 signaling in endothelium-cardiomyocyte cross-talk. J. Biol. Chem. 281 (28), 19469–19477. 10.1074/jbc.M600399200 16698793

[B114] Li B. Zheng Z. Wei Y. Wang M. Peng J. Kang T. (2011). Therapeutic effects of neuregulin-1 in diabetic cardiomyopathy rats. Cardiovasc. Diabetol. 10, 69. 10.1186/1475-2840-10-69 21798071 PMC3170868

[B115] Li J. Gu X. h. Duan J. c. Zeng L. Li Y. Wang L. (2007). [Effects of recombined human neuregulin on the contractibility of cardiac muscles of rhesus monkeys with pacing-induced heart failure]. Sichuan Da Xue Xue Bao Yi Xue Ban. 38 (1), 105–108. 17294740

[B116] Li L. Cleary S. Mandarano M. A. Long W. Birchmeier C. Jones F. E. (2002). The breast proto-oncogene, HRGalpha regulates epithelial proliferation and lobuloalveolar development in the mouse mammary gland. Oncogene 21 (32), 4900–4907. 10.1038/sj.onc.1205634 12118369

[B117] Li T. Liang W. Xiao X. Qian Y. (2018). Nanotechnology, an alternative with promising prospects and advantages for the treatment of cardiovascular diseases. Int. J. Nanomedicine 13, 7349–7362. 10.2147/IJN.S179678 30519019 PMC6233477

[B118] Li X. Zhang Y. Ren X. Wang Y. Chen D. Li Q. (2021). Ischemic microenvironment-responsive therapeutics for cardiovascular diseases. Adv. Mat. 33 (52), e2105348. 10.1002/adma.202105348 34623714

[B119] Liang X. Ding Y. Lin F. Zhang Y. Zhou X. Meng Q. (2019). Overexpression of ERBB4 rejuvenates aged mesenchymal stem cells and enhances angiogenesis via PI3K/AKT and MAPK/ERK pathways. Faseb J. 33 (3), 4559–4570. 10.1096/fj.201801690R 30566395

[B120] Lin Y. Liu H. Wang X. (2020). Neuregulin-1, a microvascular endothelial-derived protein, protects against myocardial ischemia-reperfusion injury (Review). Int. J. Mol. Med. 46 (3), 925–935. 10.3892/ijmm.2020.4662 32705151

[B121] Lin Z. Pu W. T. (2014). Harnessing Hippo in the heart: Hippo/Yap signaling and applications to heart regeneration and rejuvenation. Stem Cell Res. 13 (3), 571–581. 10.1016/j.scr.2014.04.010 24881775 PMC4223001

[B122] Lin Z. Zhou P. von Gise A. Gu F. Ma Q. Chen J. (2015). Pi3kcb links Hippo-YAP and PI3K-AKT signaling pathways to promote cardiomyocyte proliferation and survival. Circ. Res. 116 (1), 35–45. 10.1161/CIRCRESAHA.115.304457 25249570 PMC4282610

[B123] Lindsey M. L. Bolli R. Canty J. M. Du X. J. Frangogiannis N. G. Frantz S. (2018). Guidelines for experimental models of myocardial ischemia and infarction. Am. J. Physiol. Heart Circ. Physiol. 314 (4), H812–H838. 10.1152/ajpheart.00335.2017 29351451 PMC5966768

[B124] Liu F. F. Stone J. R. Schuldt A. J. T. Okoshi K. Okoshi M. P. Nakayama M. (2005). Heterozygous knockout of neuregulin-1 gene in mice exacerbates doxorubicin-induced heart failure. Am. J. Physiol. Heart Circ. Physiol. 289 (2), H660–H666. 10.1152/ajpheart.00268.2005 15833803

[B125] Liu H. Liu R. Xiong Y. Li X. Wang X. Ma Y. (2014). Leucine facilitates the insulin-stimulated glucose uptake and insulin signaling in skeletal muscle cells: Involving mTORC1 and mTORC2. Amino Acids 46 (8), 1971–1979. 10.1007/s00726-014-1752-9 24806638

[B126] Liu H. Weng X. J. Yao J. Y. Zheng J. Lv X. Zhou X. H. (2020). Neuregulin-1β protects the rat diaphragm during sepsis against oxidative stress and inflammation by activating the PI3K/Akt pathway. Oxid. Med. Cell. Longev. 2020, 1720961. 10.1155/2020/1720961 32765805 PMC7387979

[B127] Liu J. Bressan M. Hassel D. Huisken J. Staudt D. Kikuchi K. (2010). A dual role for ErbB2 signaling in cardiac trabeculation. Development 137 (22), 3867–3875. 10.1242/dev.053736 20978078 PMC3049280

[B128] Liu X. Gu X. Li Z. Li X. Li H. Chang J. (2006). Neuregulin-1/erbB-activation improves cardiac function and survival in models of ischemic, dilated, and viral cardiomyopathy. J. Am. Coll. Cardiol. 48 (7), 1438–1447. 10.1016/j.jacc.2006.05.057 17010808

[B129] Liu X. Hwang H. Cao L. BucklandM. CunninghAm A. Chen J. (1998). Domain-specific gene disruption reveals critical regulation of neuregulin signaling by its cytoplasmic tail. Proc. Natl. Acad. Sci. U. S. A. 95 (22), 13024–13029. 10.1073/pnas.95.22.13024 9789034 PMC23694

[B130] Loeb J. A. Susanto E. T. Fischbach G. D. (1998). The neuregulin precursor proARIA is processed to ARIA after expression on the cell surface by a protein kinase C-enhanced mechanism. Mol. Cell. Neurosci. 11 (1-2), 77–91. 10.1006/mcne.1998.0676 9608535

[B131] Lu F. Wei L. Yang C. Qiao Y. Liu Y. S. Chen X. D. (2021). Nrg1/ErbB2 regulates differentiation and apoptosis of neural stem cells in the cochlear nucleus through PI3K/Akt pathway. Neurosci. Lett. 751, 135803. 10.1016/j.neulet.2021.135803 33705930

[B132] Ma H. Yin C. Zhang Y. Qian L. Liu J. (2016). ErbB2 is required for cardiomyocyte proliferation in murine neonatal hearts. Gene 592 (2), 325–330. 10.1016/j.gene.2016.07.006 27390088 PMC5344651

[B133] Mahiny-Shahmohammady D. Hauck L. Billia F. (2022). Defining the molecular underpinnings controlling cardiomyocyte proliferation. Clin. Sci. 136 (12), 911–934. 10.1042/CS20211180 35723259

[B134] Marchionni M. A. Goodearl A. D. Chen M. S. Bermingham-McDOnOgh O. Kirk C. HendricksM. (1993). Glial growth factors are alternatively spliced erbB2 ligands expressed in the nervous system. Nature 362 (6418), 312–318. 10.1038/362312a0 8096067

[B135] Matsukawa R. Hirooka Y. Ito K. Sunagawa K. (2013). Inhibition of neuregulin-1/ErbB signaling in the rostral ventrolateral medulla leads to hypertension through reduced nitric oxide synthesis. Am. J. Hypertens. 26 (1), 51–57. 10.1093/ajh/hps005 23382327

[B136] Matsushima S. Tsutsui H. Sadoshima J. (2014). Physiological and pathological functions of NADPH oxidases during myocardial ischemia-reperfusion. Trends cardiovasc. Med. 24 (5), 202–205. 10.1016/j.tcm.2014.03.003 24880746 PMC4119873

[B137] Mei M. Kim Y. Sutherland L. B. Murakami M. Qi X. McAnally J. (2013). Hippo pathway effector Yap promotes cardiac regeneration. Proc. Natl. Acad. Sci. U. S. A. 110 (34), 13839–13844. 10.1073/pnas.1313192110 23918388 PMC3752208

[B138] Mei L. Nave K. A. (2014). Neuregulin-ERBB signaling in the nervous system and neuropsychiatric diseases. Neuron 83 (1), 27–49. 10.1016/j.neuron.2014.06.007 24991953 PMC4189115

[B139] Mei L. Xiong W. C. (2008). Neuregulin 1 in neural development, synaptic plasticity and schizophrenia. Nat. Rev. Neurosci. 9 (6), 437–452. 10.1038/nrn2392 18478032 PMC2682371

[B140] Mendes-Ferreira P. De Keulenaer G. W. Leite-Moreira A. F. Bras-Silva C. (2013). Therapeutic potential of neuregulin-1 in cardiovascular disease. Drug Discov. Today 18 (17-18), 836–842. 10.1016/j.drudis.2013.01.010 23384772

[B141] Mendes-Ferreira P. Maia-Rocha C. Adao R. Mendes M. J. Santos-Ribeiro D. Alves B. S. (2016). Neuregulin-1 improves right ventricular function and attenuates experimental pulmonary arterial hypertension. Cardiovasc. Res. 109 (1), 44–54. 10.1093/cvr/cvv244 26503987

[B142] Meng D. Pan H. Chen Y. Ding J. Dai Y. (2021). Roles and mechanisms of NRG1 in modulating the pathogenesis of NAFLD through ErbB3 signaling in hepatocytes (NRG1 modulates NAFLD through ErbB3 signaling). Obes. Res. Clin. Pract. 15 (2), 145–151. 10.1016/j.orcp.2021.01.003 33541789

[B143] Meyer D. Birchmeier C. (1995). Multiple essential functions of neuregulin in development. Nature 378 (6555), 386–390. 10.1038/378386a0 7477375

[B144] Meyer D. Yamaai T. GArrAtt A. RiEthmachEr-SonnEnbErg E. Kane D. Theill L. E. (1997). Isoform-specific expression and function of neuregulin. Development 124 (18), 3575–3586. 10.1242/dev.124.18.3575 9342050

[B145] Minamino T. Komuro I. (2008). Vascular aging: Insights from studies on cellular senescence, stem cell aging, and progeroid syndromes. Nat. Clin. Pract. Cardiovasc. Med. 5 (10), 637–648. 10.1038/ncpcardio1324 18762784

[B146] Minamino T. Komuro I. (2007). Vascular cell senescence: Contribution to atherosclerosis. Circ. Res. 100 (1), 15–26. 10.1161/01.RES.0000256837.40544.4a 17204661

[B147] Montero J. C. Yuste L. Diaz-RodriguEz E. EspAris-OgAndo A. PAndiellA A. (2000). Differential shedding of transmembrane neuregulin isoforms by the tumor necrosis factor-alpha-converting enzyme. Mol. Cell. Neurosci. 16 (5), 631–648. 10.1006/mcne.2000.0896 11083924

[B148] Moon J. S. Nakahira K. Chung K. P. DeNicola G. M. Koo M. J. Pabon M. A. (2016). NOX4-dependent fatty acid oxidation promotes NLRP3 inflammasome activation in macrophages. Nat. Med. 22 (9), 1002–1012. 10.1038/nm.4153 27455510 PMC5204248

[B149] Nahrendorf M. Pittet M. J. Swirski F. K. (2010). Monocytes: Protagonists of infarct inflammation and repair after myocardial infarction. Circulation 121 (22), 2437–2445. 10.1161/CIRCULATIONAHA.109.916346 20530020 PMC2892474

[B150] Nakano-Kurimoto R. Ikeda K. Uraoka M. Nakagawa Y. Yutaka K. Koide M. (2009). Replicative senescence of vascular smooth muscle cells enhances the calcification through initiating the osteoblastic transition. Am. J. Physiol. Heart Circ. Physiol. 297 (5), H1673–H1684. 10.1152/ajpheart.00455.2009 19749165

[B151] Nakaoku T. Tsuta K. Ichikawa H. Shiraishi K. Sakamoto H. Enari M. (2014). Druggable oncogene fusions in invasive mucinous lung adenocarcinoma. Clin. Cancer Res. 20 (12), 3087–3093. 10.1158/1078-0432.CCR-14-0107 24727320 PMC6329293

[B152] Nonn L. Williams R. R. Erickson R. P. Powis G. (2003). The absence of mitochondrial thioredoxin 2 causes massive apoptosis, exencephaly, and early embryonic lethality in homozygous mice. Mol. Cell. Biol. 23 (3), 916–922. 10.1128/mcb.23.3.916-922.2003 12529397 PMC140716

[B153] Odashima M. Usui S. Takagi H. Hong C. Liu J. Yokota M. (2007). Inhibition of endogenous Mst1 prevents apoptosis and cardiac dysfunction without affecting cardiac hypertrophy after myocardial infarction. Circ. Res. 100 (9), 1344–1352. 10.1161/01.RES.0000265846.23485.7a 17395874

[B154] Odiete O. Hill M. F. Sawyer D. B. (2012). Neuregulin in cardiovascular development and disease. Circ. Res. 111 (10), 1376–1385. 10.1161/CIRCRESAHA.112.267286 23104879 PMC3752394

[B155] Odiete O. Konik E. A. Sawyer D. B. Hill M. F. (2013). Type 1 diabetes mellitus abrogates compensatory augmentation of myocardial neuregulin-1β/ErbB in response to myocardial infarction resulting in worsening heart failure. Cardiovasc. Diabetol. 12, 52. 10.1186/1475-2840-12-52 23530877 PMC3617023

[B156] Okoshi K. Nakayama M. Yan X. Okoshi M. P. Schuldt A. J. T. Marchionni M. A. (2004). Neuregulins regulate cardiac parasympathetic activity: Muscarinic modulation of beta-adrenergic activity in myocytes from mice with neuregulin-1 gene deletion. Circulation 110 (6), 713–717. 10.1161/01.CIR.0000138109.32748.80 15289373

[B157] Olayioye M. A. Neve R. M. Lane H. A. Hynes N. E. (2000). The ErbB signaling network: Receptor heterodimerization in development and cancer. Embo J. 19 (13), 3159–3167. 10.1093/emboj/19.13.3159 10880430 PMC313958

[B158] Opie L. H. (1976). Effects of regional ischemia on metabolism of glucose and fatty acids. Relative rates of aerobic and anaerobic energy production during myocardial infarction and comparison with effects of anoxia. Circ. Res. 38 (5), I52–I74. 5202

[B159] Ozcelik C. Erdmann B. Pilz B. Wettschureck N. Britsch S. Hubner N. (2002). Conditional mutation of the ErbB2 (HER2) receptor in cardiomyocytes leads to dilated cardiomyopathy. Proc. Natl. Acad. Sci. U. S. A. 99 (13), 8880–8885. 10.1073/pnas.122249299 12072561 PMC124392

[B160] Parodi E. M. Kuhn B. (2014). Signalling between microvascular endothelium and cardiomyocytes through neuregulin. Cardiovasc. Res. 102 (2), 194–204. 10.1093/cvr/cvu021 24477642 PMC3989448

[B161] Peles E. Bacus S. S. Koski R. A. Lu H. S. Wen D. Ogden S. G. (1992). Isolation of the neu/HER-2 stimulatory ligand: A 44 kd glycoprotein that induces differentiation of mammary tumor cells. Cell 69 (1), 205–216. 10.1016/0092-8674(92)90131-u 1348215

[B162] Pentassuglia L. Heim P. Lebboukh S. Morandi C. Xu L. Brink M. (2016). Neuregulin-1β promotes glucose uptake via PI3K/Akt in neonatal rat cardiomyocytes. Am. J. Physiol. Endocrinol. Metab. 310 (9), E782–E794. 10.1152/ajpendo.00259.2015 26979522

[B163] Pilz P. M. Hamza O. Gidlof O. Goncalves I. F. Tretter E. V. Trojanek S. (2019). Remote ischemic perconditioning attenuates adverse cardiac remodeling and preserves left ventricular function in a rat model of reperfused myocardial infarction. Int. J. Cardiol. 285, 72–79. 10.1016/j.ijcard.2019.03.003 30904281

[B164] Pinkas-Kramarski R. ShellyM. Guarino B. C. Wang L. M. Lyass L. Alroy I. (1998). ErbB tyrosine kinases and the two neuregulin families constitute a ligand-receptor network. Mol. Cell. Biol. 18 (10), 6090–6101. 10.1128/mcb.18.10.6090 9742126 PMC109195

[B165] Plowman G. D. Green J. M. Culouscou J. M. Carlton G. W. Rothwell V. M. Buckley S. (1993). Heregulin induces tyrosine phosphorylation of HER4/p180erbB4. Nature 366 (6454), 473–475. 10.1038/366473a0 7902537

[B166] Polizzotti B. D. Ganapathy B. Walsh S. Choudhury S. Ammanamanchi N. Bennett D. G. (2015). Neuregulin stimulation of cardiomyocyte regeneration in mice and human myocardium reveals a therapeutic window. Sci. Transl. Med. 7 (281), 281ra45. 10.1126/scitranslmed.aaa5171 PMC5360874 25834111

[B167] Prabhu S. D. Frangogiannis N. G. (2016). The biological basis for cardiac repair after myocardial infarction: From inflammation to fibrosis. Circ. Res. 119 (1), 91–112. 10.1161/CIRCRESAHA.116.303577 27340270 PMC4922528

[B168] Prenzel N. Zwick E. Daub H. LesererM. AbRaham R. WallasCh C. (1999). EGF receptor transactivation by G-protein-coupled receptors requires metalloproteinase cleavage of proHB-EGF. Nature 402 (6764), 884–888. 10.1038/47260 10622253

[B169] Rebouças J. S. Santos-Magalhães N. S. Formiga F. R. (2016). Cardiac regeneration using growth factors: Advances and challenges. Arq. Bras. Cardiol. 107 (3), 271–275. 10.5935/abc.20160097 27355588 PMC5053196

[B170] Rentschler S. Zander J. Meyers K. France D. Levine R. Porter G. (2002). Neuregulin-1 promotes formation of the murine cardiac conduction system. Proc. Natl. Acad. Sci. U. S. A. 99 (16), 10464–10469. 10.1073/pnas.162301699 12149465 PMC124940

[B171] Riehle C. Abel E. D. (2016). Insulin signaling and heart failure. Circ. Res. 118 (7), 1151–1169. 10.1161/CIRCRESAHA.116.306206 27034277 PMC4833475

[B172] Rupert C. E. Coulombe K. L. (2015). The roles of neuregulin-1 in cardiac development, homeostasis, and disease. Biomark. Insights 10 (1), 1–9. 10.4137/BMI.S20061 PMC4395047 25922571

[B173] Russell K. S. Kalinowski A. Hedhli N. (2014). Cardiovascular effects of neuregulin-1/ErbB signaling: Role in vascular signaling and angiogenesis. Curr. Pharm. Des. 20 (30), 4899–4905. 10.2174/1381612819666131125151058 24283954

[B174] Safa R. N. Peng X. Y. Pentassuglia L. Lim C. C. Lamparter M. Silverstein C. (2011). Neuregulin-1β regulation of embryonic endothelial progenitor cell survival. Am. J. Physiol. Heart Circ. Physiol. 300 (4), H1311–H1319. 10.1152/ajpheart.01104.2009 21239627 PMC3075022

[B175] Sandanger Ø. Ranheim T. Vinge L. E. Bliksoen M. Alfsnes K. Finsen A. V. (2013). The NLRP3 inflammasome is up-regulated in cardiac fibroblasts and mediates myocardial ischaemia-reperfusion injury. Cardiovasc. Res. 99 (1), 164–174. 10.1093/cvr/cvt091 23580606

[B176] Sarkar S. Korolchuk V. I. Renna M. Imarisio S. Fleming A. Williams A. (2011). Complex inhibitory effects of nitric oxide on autophagy. Mol. Cell 43 (1), 19–32. 10.1016/j.molcel.2011.04.029 21726807 PMC3149661

[B177] Sato M. Dehvari N. Oberg A. I. Dallner O. S. Sandstrom A. L. Olsen J. M. (2014). Improving type 2 diabetes through a distinct adrenergic signaling pathway involving mTORC2 that mediates glucose uptake in skeletal muscle. Diabetes 63 (12), 4115–4129. 10.2337/db13-1860 25008179

[B178] Sawyer D. B. Zuppinger C. Miller T. A. Eppenberger H. M. Suter T. M. (2002). Modulation of anthracycline-induced myofibrillar disarray in rat ventricular myocytes by neuregulin-1beta and anti-erbB2: Potential mechanism for trastuzumab-induced cardiotoxicity. Circulation 105 (13), 1551–1554. 10.1161/01.cir.0000013839.41224.1c 11927521

[B179] Saxton R. A. Sabatini D. M. (2017). mTOR signaling in growth, metabolism, and disease. Cell 168 (6), 960–976. 10.1016/j.cell.2017.02.004 28283069 PMC5394987

[B180] Schaefer M. Plant T. D. Obukhov A. G. Hofmann T. Gudermann T. Schultz G. (2000). Receptor-mediated regulation of the nonselective cation channels TRPC4 and TRPC5. J. Biol. Chem. 275 (23), 17517–17526. 10.1074/jbc.275.23.17517 10837492

[B181] Schwach V. Gomes Fernandes M. Maas S. Gerhardt S. Tsonaka R. van der Weerd L. (2020). Expandable human cardiovascular progenitors from stem cells for regenerating mouse heart after myocardial infarction. Cardiovasc. Res. 116 (3), 545–553. 10.1093/cvr/cvz181 31287499 PMC7252440

[B182] Seidman J. G. Seidman C. (2001). The genetic basis for cardiomyopathy: From mutation identification to mechanistic paradigms. Cell 104 (4), 557–567. 10.1016/s0092-8674(01)00242-2 11239412

[B183] Shakeri H. Boen J. R. A. De Moudt S. Hendrickx J. O. Leloup A. J. A. Jacobs G. (2021). Neuregulin-1 compensates for endothelial nitric oxide synthase deficiency. Am. J. Physiol. Heart Circ. Physiol. 320 (6), H2416–h2428. 10.1152/ajpheart.00914.2020 33989083

[B184] Shakeri H. Gevaert A. B. Schrijvers D. M. De Meyer G. R. Y. De Keulenaer G. W. Guns P. J. D. F. (2018). Neuregulin-1 attenuates stress-induced vascular senescence. Cardiovasc. Res. 114 (7), 1041–1051. 10.1093/cvr/cvy059 29528383

[B185] Shao D. Tian R. (2015). Glucose transporters in cardiac metabolism and hypertrophy. Compr. Physiol. 6 (1), 331–351. 10.1002/cphy.c150016 26756635 PMC4760112

[B186] Shende P. Plaisance I. Morandi C. Pellieux C. Berthonneche C. Zorzato F. (2011). Cardiac raptor ablation impairs adaptive hypertrophy, alters metabolic gene expression, and causes heart failure in mice. Circulation 123 (10), 1073–1082. 10.1161/CIRCULATIONAHA.110.977066 21357822

[B187] Shi W. Chen H. Sun J. Buckley S. Zhao J. Anderson K. D. (2003). TACE is required for fetal murine cardiac development and modeling. Dev. Biol. 261 (2), 371–380. 10.1016/s0012-1606(03)00315-4 14499647

[B188] Shiraishi M. Yamaguchi A. Suzuki K. (2022). Nrg1/ErbB signaling-mediated regulation of fibrosis after myocardial infarction. Faseb J. 36 (2), e22150. 10.1096/fj.202101428RR 34997943

[B189] Shirakabe K. WakatSuki S. Kurisaki T. FujisAwA-SehArA A. (2001). Roles of Meltrin beta/ADAM19 in the processing of neuregulin. J. Biol. Chem. 276 (12), 9352–9358. 10.1074/jbc.M007913200 11116142

[B190] Sikora E. Arendt T. Bennett M. Narita M. (2011). Impact of cellular senescence signature on ageing research. Ageing Res. Rev. 10 (1), 146–152. 10.1016/j.arr.2010.10.002 20946972

[B191] Simón-Yarza T. Rossi A. Heffels K. H. Prosper F. Groll J. Blanco-Prieto M. J. (2015). Polymeric electrospun scaffolds: Neuregulin encapsulation and biocompatibility studies in a model of myocardial ischemia. Tissue Eng. Part A 21 (9-10), 1654–1661. 10.1089/ten.TEA.2014.0523 25707939

[B192] Smits A. M. Dronkers E. Goumans M. J. (2018). The epicardium as a source of multipotent adult cardiac progenitor cells: Their origin, role and fate. Pharmacol. Res. 127, 129–140. 10.1016/j.phrs.2017.07.020 28751220

[B193] Staudt D. W. Liu J. Thorn K. S. Stuurman N. Liebling M. Stainier D. Y. R. (2014). High-resolution imaging of cardiomyocyte behavior reveals two distinct steps in ventricular trabeculation. Development 141 (3), 585–593. 10.1242/dev.098632 24401373 PMC3899815

[B194] Steffens S. Van Linthout S. Sluijter J. P. G. Tocchetti C. G. Thum T. Madonna R. (2020). Stimulating pro-reparative immune responses to prevent adverse cardiac remodelling: Consensus document from the joint 2019 meeting of the ESC working groups of cellular biology of the heart and myocardial function. Cardiovasc. Res. 116 (11), 1850–1862. 10.1093/cvr/cvaa137 32396608

[B195] Steinthorsdottir V. Stefansson H. Ghosh S. Birgisdottir B. Bjornsdottir S. Fasquel A. C. (2004). Multiple novel transcription initiation sites for NRG1. Gene 342 (1), 97–105. 10.1016/j.gene.2004.07.029 15527969

[B196] Stephen M. J. Poindexter B. J. Moolman J. A. Sheikh-Hamad D. Bick R. J. (2009). Do binucleate cardiomyocytes have a role in myocardial repair? Insights using isolated rodent myocytes and cell culture. Open cardiovasc. Med. J. 3, 1–7. 10.2174/1874192400903010001 19430572 PMC2678822

[B197] Stonecypher M. S. Chaudhury A. R. Byer S. J. Carroll S. L. (2006). Neuregulin growth factors and their ErbB receptors form a potential signaling network for schwannoma tumorigenesis. J. Neuropathol. Exp. Neurol. 65 (2), 162–175. 10.1097/01.jnen.0000199575.93794.2f 16462207

[B198] Sudol M. (2014). Neuregulin 1-activated ERBB4 as a "dedicated" receptor for the Hippo-YAP pathway. Sci. Signal. 7 (355), pe29. 10.1126/scisignal.aaa2710 25492964

[B199] Suk Kim H. Hidaka K. Morisaki T. (2003). Expression of ErbB receptors in ES cell-derived cardiomyocytes. Biochem. Biophys. Res. Commun. 309 (1), 241–246. 10.1016/s0006-291x(03)01521-3 12943688

[B200] Sun K. Li Y. Y. Jin J. (2021). A double-edged sword of immuno-microenvironment in cardiac homeostasis and injury repair. Signal Transduct. Target. Ther. 6 (1), 79. 10.1038/s41392-020-00455-6 33612829 PMC7897720

[B201] Surviladze Z. Sterk R. T. DeHaro S. A. Ozbun M. A. (2013). Cellular entry of human papillomavirus type 16 involves activation of the phosphatidylinositol 3-kinase/Akt/mTOR pathway and inhibition of autophagy. J. Virol. 87 (5), 2508–2517. 10.1128/JVI.02319-12 23255786 PMC3571372

[B202] Sweeney C. Fambrough D. Huard C. Diamonti A. J. Lander E. S. Cantley L. C. (2001). Growth factor-specific signaling pathway stimulation and gene expression mediated by ErbB receptors. J. Biol. Chem. 276 (25), 22685–22698. 10.1074/jbc.M100602200 11297548

[B203] Szablewski L. (2017). Glucose transporters in healthy heart and in cardiac disease. Int. J. Cardiol. 230, 70–75. 10.1016/j.ijcard.2016.12.083 28034463

[B204] Tane S. Okayama H. Ikenishi A. Amemiya Y. Nakayama K. I. Takeuchi T. (2015). Two inhibitory systems and CKIs regulate cell cycle exit of mammalian cardiomyocytes after birth. Biochem. Biophys. Res. Commun. 466 (2), 147–154. 10.1016/j.bbrc.2015.08.102 26363457

[B205] Tevzadze N. Rukhadze R. Dzidziguri D. (2005). The age related changes in cell cycle of mice cardiomyocytes. Georgian Med. News 1 (128), 87–90. 16369074

[B206] Timolati F. Ott D. Pentassuglia L. Giraud M. N. Perriard J. C. Suter T. M. (2006). Neuregulin-1 beta attenuates doxorubicin-induced alterations of excitation-contraction coupling and reduces oxidative stress in adult rat cardiomyocytes. J. Mol. Cell. Cardiol. 41 (5), 845–854. 10.1016/j.yjmcc.2006.08.002 17005195

[B207] Trombetta D. Graziano P. Scarpa A. Sparaneo A. Rossi G. Rossi A. (2018). Frequent NRG1 fusions in Caucasian pulmonary mucinous adenocarcinoma predicted by Phospho-ErbB3 expression. Oncotarget 9 (11), 9661–9671. 10.18632/oncotarget.23800 29515761 PMC5839392

[B208] Vandekerckhove L. Vermeulen Z. Liu Z. Z. Boimvaser S. Patzak A. Segers V. F. M. (2016). Neuregulin-1 attenuates development of nephropathy in a type 1 diabetes mouse model with high cardiovascular risk. Am. J. Physiol. Endocrinol. Metab. 310 (7), E495–E504. 10.1152/ajpendo.00432.2015 26786778 PMC4824141

[B209] Vasti C. Witt H. Said M. Sorroche P. Garcia-Rivello H. Ruiz-Noppinger P. (2012). Doxorubicin and NRG-1/erbB4-deficiency affect gene expression profile: Involving protein homeostasis in mouse. ISRN Cardiol. 2012, 745185. 10.5402/2012/745185 22970387 PMC3437290

[B210] Vermeulen Z. Hervent A. S. Dugaucquier L. Vandekerckhove L. Rombouts M. Beyens M. (2017). Inhibitory actions of the NRG-1/ErbB4 pathway in macrophages during tissue fibrosis in the heart, skin, and lung. Am. J. Physiol. Heart Circ. Physiol. 313 (5), H934–H945. 10.1152/ajpheart.00206.2017 28822966

[B211] Vicier C. Dieci M. V. Arnedos M. Delaloge S. Viens P. Andre F. (2014). Clinical development of mTOR inhibitors in breast cancer. Breast Cancer Res. 16 (1), 203. 10.1186/bcr3618 25189767 PMC3978635

[B212] von Gise A. Lin Z. Schlegelmilch K. Honor L. B. Pan G. M. Buck J. N. (2012). YAP1, the nuclear target of Hippo signaling, stimulates heart growth through cardiomyocyte proliferation but not hypertrophy. Proc. Natl. Acad. Sci. U. S. A. 109 (7), 2394–2399. 10.1073/pnas.1116136109 22308401 PMC3289361

[B213] Wadugu B. Kühn B. (2012). The role of neuregulin/ErbB2/ErbB4 signaling in the heart with special focus on effects on cardiomyocyte proliferation. Am. J. Physiol. Heart Circ. Physiol. 302 (11), H2139–H2147. 10.1152/ajpheart.00063.2012 22427524 PMC3378290

[B214] Wagner J. U. G. Dimmeler S. (2020). Cellular cross-talks in the diseased and aging heart. J. Mol. Cell. Cardiol. 138, 136–146. 10.1016/j.yjmcc.2019.11.152 31783034

[B215] Wan A. Rodrigues B. (2016). Endothelial cell-cardiomyocyte crosstalk in diabetic cardiomyopathy. Cardiovasc. Res. 111 (3), 172–183. 10.1093/cvr/cvw159 27288009 PMC4957492

[B216] Wang F. Wang H. Liu X. Yu H. Huang X. Huang W. (2021). Neuregulin-1 alleviate oxidative stress and mitigate inflammation by suppressing NOX4 and NLRP3/caspase-1 in myocardial ischaemia-reperfusion injury. J. Cell. Mol. Med. 25 (3), 1783–1795. 10.1111/jcmm.16287 33470533 PMC7875921

[B217] Wang F. Wang H. Liu X. Yu H. Zuo B. Song Z. (2018). Pharmacological postconditioning with Neuregulin-1 mimics the cardioprotective effects of ischaemic postconditioning via ErbB4-dependent activation of reperfusion injury salvage kinase pathway. Mol. Med. 24 (1), 39. 10.1186/s10020-018-0040-7 30134819 PMC6069706

[B218] Wang J. C. Bennett M. (2012). Aging and atherosclerosis: Mechanisms, functional consequences, and potential therapeutics for cellular senescence. Circ. Res. 111 (2), 245–259. 10.1161/CIRCRESAHA.111.261388 22773427

[B219] Wang J. Y. Miller S. J. Falls D. L. (2001). The N-terminal region of neuregulin isoforms determines the accumulation of cell surface and released neuregulin ectodomain. J. Biol. Chem. 276 (4), 2841–2851. 10.1074/jbc.M005700200 11042203

[B220] Wang J. Zhou J. Wang Y. Yang C. Fu M. Zhang J. (2017). Qiliqiangxin protects against anoxic injury in cardiac microvascular endothelial cells via NRG-1/ErbB-PI3K/Akt/mTOR pathway. J. Cell. Mol. Med. 21 (9), 1905–1914. 10.1111/jcmm.13111 28271613 PMC5571527

[B221] Wang X. H. Zhuo X. Z. Ni Y. J. Gong M. Wang T. Z. Lu Q. (2012). Improvement of cardiac function and reversal of gap junction remodeling by Neuregulin-1β in volume-overloaded rats with heart failure. J. Geriatr. Cardiol. 9 (2), 172–179. 10.3724/SP.J.1263.2012.03271 22916065 PMC3418908

[B222] Wang X. Zhuo X. Gao J. Liu H. Lin F. Ma A. (2019). Neuregulin-1β partially improves cardiac function in volume-overload heart failure through regulation of abnormal calcium handling. Front. Pharmacol. 10, 616. 10.3389/fphar.2019.00616 31281251 PMC6597678

[B223] Wang Y. Y. Li T. Liu Y. W. Liu B. J. Hu X. M. (2017). [Effect of the ischemic post-conditioning on the prevention of the cardio-renal damage in patients with acute ST-segment elevation myocardial infarction after primary percutaneous coronary intervention]. Zhonghua Xin Xue Guan Bing Za Zhi 45 (4), 277–282. 10.3760/cma.j.issn.0253-3758.2017.04.005 28545277

[B224] Wang Z. Huang J. (2014). Neuregulin-1 increases connexin-40 and connexin-45 expression in embryonic stem cell-derived cardiomyocytes. Appl. Biochem. Biotechnol. 174 (2), 483–493. 10.1007/s12010-014-1089-6 25080381

[B225] Wei H. Wang C. Guo R. Takahashi K. Naruse K. (2019). Development of a model of ischemic heart disease using cardiomyocytes differentiated from human induced pluripotent stem cells. Biochem. Biophys. Res. Commun. 520 (3), 600–605. 10.1016/j.bbrc.2019.09.119 31623826

[B226] Whelan R. S. Kaplinskiy V. Kitsis R. N. (2010). Cell death in the pathogenesis of heart disease: Mechanisms and significance. Annu. Rev. Physiol. 72, 19–44. 10.1146/annurev.physiol.010908.163111 20148665 PMC12973270

[B227] Wischmeyer E. Döring F. Karschin A. (1998). Acute suppression of inwardly rectifying Kir2.1 channels by direct tyrosine kinase phosphorylation. J. Biol. Chem. 273 (51), 34063–34068. 10.1074/jbc.273.51.34063 9852063

[B228] Wu C. Gui C. Li L. Pang Y. Tang Z. Wei J. (2018). Expression and secretion of neuregulin-1 in cardiac microvascular endothelial cells treated with angiogenic factors. Exp. Ther. Med. 15 (4), 3577–3581. 10.3892/etm.2018.5811 29545886 PMC5840939

[B229] Wu J. Y. Yu H. Cohen I. S. (2000). Epidermal growth factor increases i(f) in rabbit SA node cells by activating a tyrosine kinase. Biochim. Biophys. Acta 1463 (1), 15–19. 10.1016/s0005-2736(99)00233-3 10631290

[B230] Wu L. Walas S. Leung W. Sykes D. B. Wu J. Lo E. H. (2015). Neuregulin1-β decreases IL-1β-induced neutrophil adhesion to human brain microvascular endothelial cells. Transl. Stroke Res. 6 (2), 116–124. 10.1007/s12975-014-0347-9 24863743 PMC4247352

[B231] Wu X. Reboll M. R. Korf-Klingebiel M. Wollert K. C. (2021). Angiogenesis after acute myocardial infarction. Cardiovasc. Res. 117 (5), 1257–1273. 10.1093/cvr/cvaa287 33063086

[B232] Xiao J. Li B. Zheng Z. Wang M. Peng J. Li Y. (2012). Therapeutic effects of neuregulin-1 gene transduction in rats with myocardial infarction. Coron. Artery Dis. 23 (7), 460–468. 10.1097/MCA.0b013e32835877da 22968213

[B233] Xiao Y. Hill M. C. Zhang M. Martin T. J. Morikawa Y. Wang S. (2018). Hippo signaling plays an essential role in cell state transitions during cardiac fibroblast development. Dev. Cell 45 (2), 153–169. 10.1016/j.devcel.2018.03.019 29689192 PMC5947860

[B234] Xin M. Kim Y. Sutherland L. B. Murakami M. Qi X. McAnally J. (2013). Hippo pathway effector Yap promotes cardiac regeneration. Proc. Natl. Acad. Sci. U. S. A. 110 (34), 13839–13844. 10.1073/pnas.1313192110 23918388 PMC3752208

[B235] Xin M. Kim Y. Sutherland L. B. Qi X. McAnally J. Schwartz R. J. (2011). Regulation of insulin-like growth factor signaling by yap governs cardiomyocyte proliferation and embryonic heart size. Sci. Signal. 4 (196), ra70. 10.1126/scisignal.2002278 22028467 PMC3440872

[B236] Xin M. Kim Y. Sutherland L. B. Qi X. McAnally J. Schwartz R. J. (2011). Regulation of insulin-like growth factor signaling by Yap governs cardiomyocyte proliferation and embryonic heart size. Sci. Signal. 4 (196), ra70. 10.1126/scisignal.2002278 22028467 PMC3440872

[B237] Xu G. Watanabe T. Iso Y. Koba S. Sakai T. Nagashima M. (2009). Preventive effects of heregulin-beta1 on macrophage foam cell formation and atherosclerosis. Circ. Res. 105 (5), 500–510. 10.1161/CIRCRESAHA.109.193870 19644050

[B238] Xu M. Wu X. Jie B. Zhang X. Zhang J. Xin Y. (2014). Neuregulin-1 protects myocardial cells against H2 O2 -induced apoptosis by regulating endoplasmic reticulum stress. Cell biochem. Funct. 32 (5), 464–469. 10.1002/cbf.3038 24867233

[B239] Xu Y. Li X. Liu X. Zhou M. (2010). Neuregulin-1/ErbB signaling and chronic heart failure. Adv. Pharmacol. 59, 31–51. 10.1016/S1054-3589(10)59002-1 20933198

[B240] Yamamoto S. Yang G. Zablocki D. Liu J. Hong C. Kim S. J. (2003). Activation of Mst1 causes dilated cardiomyopathy by stimulating apoptosis without compensatory ventricular myocyte hypertrophy. J. Clin. Invest. 111 (10), 1463–1474. 10.1172/JCI17459 12750396 PMC155047

[B241] Yamamoto Yang G. Zablocki D. Liu J. Hong C. Kim S. J. (2003). Activation of Mst1 causes dilated cardiomyopathy by stimulating apoptosis without compensatory ventricular myocyte hypertrophy. J. Clin. Invest. 111 (10), 1463–1474. 10.1172/JCI17459 12750396 PMC155047

[B242] Yarden Y. Sliwkowski M. X. (2001). Untangling the ErbB signalling network. Nat. Rev. Mol. Cell Biol. 2 (2), 127–137. 10.1038/35052073 11252954

[B243] Yiadom M. Y. Greenberg J. Smith H. M. Sawyer D. B. Liu D. Carlise J. (2016). Diagnostic utility of neuregulin for acute coronary syndrome. Dis. Markers 2016, 8025271. 10.1155/2016/8025271 27110055 PMC4823486

[B244] Yin D. M. Chen Y. J. Liu S. Jiao H. Shen C. SAthyAmurthy A. (2015). Calcyon stimulates neuregulin 1 maturation and signaling. Mol. Psychiatry 20 (10), 1251–1260. 10.1038/mp.2014.131 25349163

[B245] Yin H. Favreau-Lessard A. J. deKay J. T. Herrmann Y. R. Robich M. P. Koza R. A. (2021). Protective role of ErbB3 signaling in myeloid cells during adaptation to cardiac pressure overload. J. Mol. Cell. Cardiol. 152, 1–16. 10.1016/j.yjmcc.2020.11.009 33259856 PMC7981250

[B246] Zhang D. Contu R. Latronico M. V. G. Zhang J. Zhang J. L. Rizzi R. (2010). MTORC1 regulates cardiac function and myocyte survival through 4E-BP1 inhibition in mice. J. Clin. Invest. 120 (8), 2805–2816. 10.1172/JCI43008 20644257 PMC2912201

[B247] Zhang J. Ji J. Y. Yu M. Overholtzer M. Smolen G. A. Wang R. (2009). YAP-dependent induction of amphiregulin identifies a non-cell-autonomous component of the Hippo pathway. Nat. Cell Biol. 11 (12), 1444–1450. 10.1038/ncb1993 19935651 PMC2819909

[B248] Zhang P. Kuang H. He Y. Idiga S. O. Li S. Chen Z. (2018). NRG1-Fc improves metabolic health via dual hepatic and central action. JCI Insight 3 (5), 98522. 10.1172/jci.insight.98522 29515030 PMC5922292

[B249] Zhang Y. Re D. D. (2017). A growing role for the Hippo signaling pathway in the heart. J. Mol. Med. 95 (5), 465–472. 10.1007/s00109-017-1525-5 28280861 PMC5404975

[B250] Zhao W. J. Schachner M. (2013). Neuregulin 1 enhances cell adhesion molecule l1 expression in human glioma cells and promotes their migration as a function of malignancy. J. Neuropathol. Exp. Neurol. 72 (3), 244–255. 10.1097/NEN.0b013e3182863dc5 23399902

[B251] Zhao Y. Y. FerOn O. Dessy C. Han X. Marchionni M. A. Kelly R. A. (1999). Neuregulin signaling in the heart. Dynamic targeting of erbB4 to caveolar microdomains in cardiac myocytes. Circ. Res. 84 (12), 1380–1387. 10.1161/01.res.84.12.1380 10381889

[B252] Zhao Y. Y. Sawyer D. R. Baliga R. R. Opel D. J. Han X. Marchionni M. A. (1998). Neuregulins promote survival and growth of cardiac myocytes. Persistence of ErbB2 and ErbB4 expression in neonatal and adult ventricular myocytes. J. Biol. Chem. 273 (17), 10261–10269. 10.1074/jbc.273.17.10261 9553078

[B253] Zhu W. Z. Xie Y. Moyes K. W. Gold J. D. Askari B. Laflamme M. A. (2010). Neuregulin/ErbB signaling regulates cardiac subtype specification in differentiating human embryonic stem cells. Circ. Res. 107 (6), 776–786. 10.1161/CIRCRESAHA.110.223917 20671236 PMC2941561

[B254] Zurek M. Johansson E. Palmer M. Albery T. Johansson K. Ryden-Markinhutha K. (2020). Neuregulin-1 induces cardiac hypertrophy and impairs cardiac performance in post-myocardial infarction rats. Circulation 142 (13), 1308–1311. 10.1161/CIRCULATIONAHA.119.044313 32986480

